# Design and screening of novel endosomal escape compounds that enhance functional delivery of oligonucleotides *in vitro*

**DOI:** 10.1016/j.omtn.2025.102522

**Published:** 2025-03-20

**Authors:** H. Yesid Estupiñán, Tom Baladi, Samantha Roudi, Michael J. Munson, Jeremy Bost, Oskar Gustafsson, Daniel Velásquez-Ramírez, Deepak Kumar Bhatt, Daniel Hagey, Dennis Hekman, Shalini Andersson, Samir EL Andaloussi, Anders Dahlén

**Affiliations:** 1 Department of Laboratory Medicine, Karolinska Institutet, ANA Futura, Huddinge, Sweden; 2 Departamento de Ciencias Básicas, Universidad Industrial de Santander, Bucaramanga, Colombia; 3 Karolinska ATMP Center, ANA Futura, Huddinge, Sweden; 4 Department of Cellular Therapy and Allogeneic Stem Cell Transplantation (CAST), Karolinska University Hospital, Huddinge, Sweden; 5 Discovery Sciences, BioPharmaceuticals R&D, AstraZeneca, Gothenburg, Sweden; 6 Advanced Drug Delivery, Pharmaceutical Sciences, BioPharmaceuticals R&D, AstraZeneca, Mölndal, Sweden; 7 DMPK, Research and Early Development Cardiovascular Renal and Metabolism, BioPharmaceuticals R&D, AstraZeneca, Gothenburg, Sweden

**Keywords:** MT: Oligonucleotides: Therapies and Applications, oligonucleotides, antisense, therapeutic, small molecules, enhancers, endosomes, lysosomes, leakage, mitophagy

## Abstract

Antisense oligonucleotides (ASOs), including splice-switching oligonucleotides (SSOs), are promising therapeutic approaches for targeting genetic defects. ASOs act in the nucleus and the cytosol to cleave mRNAs via the RNaseH1 mechanism (e.g., gapmers), while SSOs alter transcript splicing to restore or inhibit protein function. RNA interference (RNAi) is another approach to down-regulate gene expression via the RISC complex. However, a major challenge is the effective delivery of these nucleic acid-based therapeutics. Recent developments focus on enhancing cellular uptake and endosomal release, including the use of small-molecule endosomal escape enhancers (EEEs) such as chloroquine. Here, we disclose a next generation of EEEs, which efficiently enhance SSOs and gapmers *in vitro* activity. We identify proton sponge-mediated endosomal leakage as a mechanism of action and observe, by Gene Ontology analysis on bulk RNA sequencing, that EEE treatment increased gene expression of markers associated with vesicle organization. Additionally, using primary human hepatocytes, we demonstrate that EEEs enhance small interfering RNA (siRNA) activity. Unconjugated siRNA reached similar levels of mRNA knockdown to the observed GalNAc-conjugated siRNA. Substantial GalNAc conjugated siRNA enhancement was also observed when used together with EEE. Our results indicate that these EEEs constitute a promising strategy to enhance the activity of multimodal oligonucleotide therapeutics.

## Graphical abstract

**Figure:**
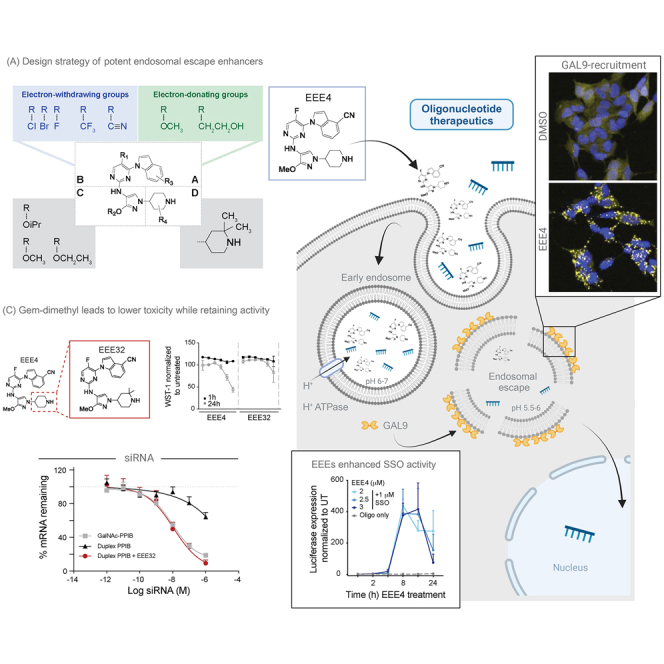

## Teaser

Structure activity relationship studies by Estupiñán and colleagues led to the design of potent endosomal escape enhancers (EEEs) that increase splice-switching oligonucleotide activity up to 400-fold over control. Additional studies measuring long non-coding RNA and mRNA knockdown demonstrate EEEs potential as enhancers of ASO gapmers, naked small interfering RNA (siRNA) and GalNAc-conjugated siRNA.

## Introduction

Oligonucleotide therapeutics represent an expanding strategy to treat a wide spectrum of diseases, with the initial focus on gene silencing, but now they also hold great potential for splice modulation and gene activation. Antisense oligonucleotides (ASOs) are short, synthetic, single-stranded DNA or RNA molecules that are designed to selectively bind to a specific target messenger RNA (mRNA) or other nucleic acid sequences (e.g., unspliced pre-mRNA). These ASOs are complementary to the targeted RNA sequence and by binding, they can modulate gene expression through various mechanisms. Many therapeutic ASOs are so-called gapmers, with a central DNA region flanked by chemically modified ends to increase affinity and reduce susceptibility to nucleases. These gapmers promote cleavage of mRNAs by activating the RNase H1-dependent mechanism in a catalytic fashion. Another type of ASOs is splice-switching oligonucleotides (SSOs), which are designed to bind pre-mRNA to promote or inhibit the recruitment of splicing factors. SSOs modify transcript alternative splicing by altering splice site recognition and exon structure or by inducing exon inclusion or skipping, thereby modulating protein function by producing alternative protein isoforms.

Splice-switching is a promising therapeutic approach for rare diseases like Duchenne muscular dystrophy (DMD) and spinal muscular atrophy (SMA).^1^^,^^2^^,^^3^^,^^4^^,^^5^^,^^6^^,^^7^ In the case of DMD, the currently approved SSOs are designed to selectively skip *DMD* frame-shifting exons to restore the reading frame of the dystrophin transcript, generating an exon-skipped transcript but partially functional protein.^8^ In SMA, the therapeutic strategy is to compensate the insufficient expression of survival motor neuron (SMN) protein caused by the loss-of-function mutations in the *SMN1* gene. SSOs are used to restore a splice-site defect in the paralogous *SMN2* gene promoting the inclusion of the initially skipped exon 7, thereby restoring *SMN2* mRNA and generating more SMN protein.^9^^,^^10^

Small interfering RNA (siRNA) is a class of double-stranded RNA molecules typically around 19–23 bp in length. This type of oligonucleotide is involved in the RNA interference (RNAi) pathway, an endogenous regulatory mechanism for controlling gene expression. When introduced into a cell, siRNAs are loaded into Argonaut-2 (AGO2) which is part of a protein complex called the RNA-induced silencing complex (RISC). Within RISC, the siRNA unwinds and discards the passenger strand, while the other strand known as the guide strand, stays associated with AGO2 to direct and bind complementary mRNA sequences and finally cleave the mRNA. The downstream effects of both gapmer-type ASOs and siRNAs are therefore downregulation of protein levels.^1^^,^^2^^,^^11^^,^^12^

Despite having tremendous therapeutic potential, nucleotide sequestration in the endosome and poor release into the cytosol remain a common challenge for both single- and double-stranded oligonucleotides, thus limiting the potential of oligonucleotide therapeutics. Many approaches focus on enhancing the uptake into cells using targeting moieties (e.g., galactosamine-*N*-acetyl [GalNAc] or lipids). Although these delivery modalities drastically enhance ASO and siRNA activity, it is believed that <1.5% of internalized material is released from endosomes.^13^^,^^14^ Hence, extensive efforts have also been put into promoting more efficient endosomal escape of nucleotide therapeutics. The most common approaches to date are based on formulating oligonucleotides into nanoparticles such as lipid nanoparticles (LNPs), cationic lipoplexes (e.g., Lipofectamine 2000 [LF2000]), or cell-penetrating peptide (CPP) particles.^1^^,^^15^^,^^16^^,^^17^^,^^18^^,^^19^^,^^20^

Another strategy that has gained increasing attention is the use of endosomal escape enhancers (EEEs), small molecules that facilitate the release of entrapped cargo from endosomal compartments. The most commonly known mechanism of action for these lysosomotropic molecules is endosomal leakage induced by a proton sponge effect; however, interactions with endosomal proteins such as Retro-1 have also been identified as alternative mechanisms.^15^^,^^21^^,^^22^^,^^23^^,^^24^ Our group has previously identified two EEEs that are able to enhance SSO activity at relatively low concentrations.^15^ However, these compounds have limited applicability due to the narrowed window between oligonucleotide enhancement and compromised cell viability. Hence, in this study, we carefully optimized previously published EEEs to better understand the structure activity relationship (SAR), thereby significantly improving oligonucleotide enhancing capacity and reducing cell toxicity margins.

## Results

In this study, a total of 32 EEEs were synthesized, screened and initially assessed using the galectin-9 (GAL9) assay to quantify the extent of endosomal membrane remodeling. For this purpose, we used cell lines carrying a mCherry-GAL9-reporter, where endosomal rupture can be quantified by measuring GAL9 clustering as a puncta event, representative of endosomal remodeling. A battery of *in vitro* assays was used to assess the mechanism of action of EEEs, and *in vitro* or *ex vivo* systems were used to evaluate the activity of delivered oligonucleotide therapeutics.

### SAR studies led to the design of potent endosomal escape enhancers

**Figure 1:**
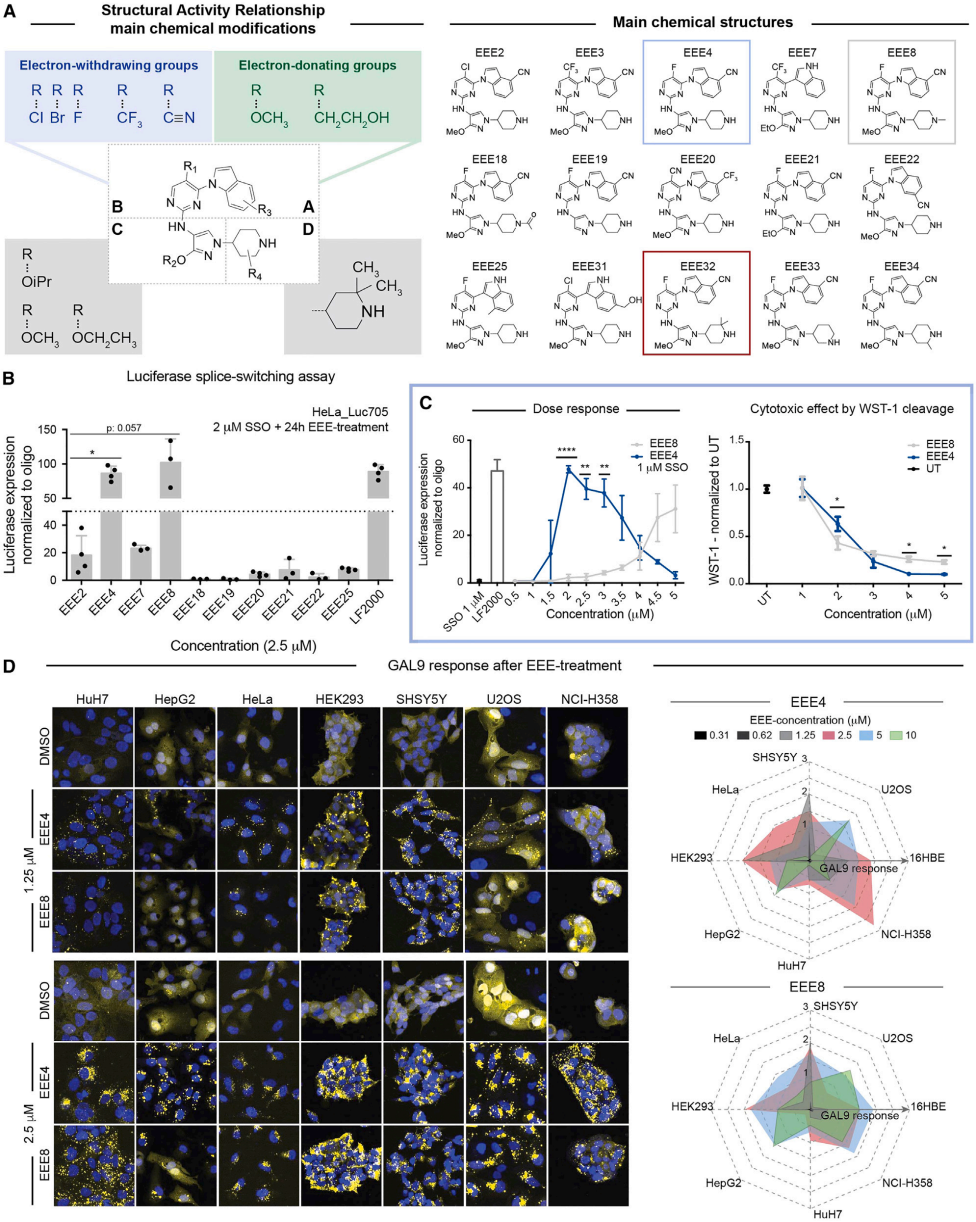
Structure activity relationship studies led to the design of new compounds that can enhance endosomal escape of SSOs (A) Main strategy and chemical substitutions used for the structure activity relationship analysis. (B) Representative EEE structures generated. The boxes highlight leading EEEs. (B and C) Screening of functional SSO delivery in HeLa_Luc705 cells. Cells were pretreated with SSOs for 24 h, followed by 24-h EEE treatment at the indicated concentration, rinsed, 0.1% Triton X-100 lysed, and luciferase intensity measured. When indicated, cell proliferation reagent WST-1 (1:10 ratio) was used, the 4-h pre-incubation step was included, and absorbance measured against background control. Bars indicate mean with SD. (D) Representative images from eight cell lines treated with EEE displaying GAL9 translocation, from cytosolic to punctate. GAL9 events are observed as puncta (yellow), and nuclei were Hoechst stained (blue); scale bars, 5 μm. GAL9 maximum quantitation is displayed as nuclei normalized signal. Significance was calculated for single variable using the Mann-Whitney *U* test and for multiple variables comparison t test using the Sidak-Bonferroni correction method (α = 0.05) (∗*p* < 0.05; ∗∗*p* < 0.005; ∗∗∗*p* < 0.0005; ∗∗∗∗*p* < 0.00005).

We investigated the SAR of two potent EEEs previously disclosed by our group.^15^ For that purpose, we studied the impact of introducing electron-withdrawing (EWG) and electron-donating groups (EDG) on each ring system of the original compounds (Figure 1A). It soon became evident that the SAR was quite narrow and that minor changes had a dramatic impact, both positive and negative.

Initially, we observed that EWG on the indole (ring A) and the pyrimidine (ring B) generally increased the potency of the molecules, while EDG lowered the effect. As observed through careful review of NMR spectra and confirmed by X-ray analysis of one of the analogs, the presence of such EWGs/EDGs dictated the regioselectivity of the coupling between the indole A and the pyrimidine B, leading to two sub-categories of EEEs. This varying regioselectivity between *N*-aryl and *C*-aryl could explain the differences in activity, as the active EEEs belong to the former sub-category (Figure 1A; Table S1). As for the pyrimidine (ring B), the SAR trend was, again, that stronger EWG induces stronger EEE activity, with fluoride being the best substituent overall. In particular, the combination of EWG on both ring A and ring B gave optimal results (Figure 1A; Tables S1 and S2). In pyrazole ring C, it was clear that small alkoxy substituents gave optimal results (i.e., Me>Et>*i*Pr). However, removing the alkoxy group altogether led to a loss of activity (Figure 1A; Tables S1 and S2). A wider chemical diversity was tolerated on the piperidine (ring D)—introducing additional alkyl substituents did not cause a loss of potency.

Although the GAL9 assay serves as a useful screening tool to assess the magnitude of endosomal rupture, it does not necessarily correlate with the enhanced activity of co-delivered ASOs. Therefore, next, we functionally screened the SSO delivery in the HeLa_Luc705 reporter cell line. HeLa_Luc705 carries a pLuc/705 splice-switching reporter luciferase gene interrupted by a mutated β-globin intron, which creates a preferentially used cryptic 5′ splice site leading to aberrant splicing. Functional SSO delivery masks the cryptic site, redirecting the splicing machinery to the right splicing site, allowing the measurement of luciferase expression (Figure S1).^25^^,^^26^^,^^27^^,^^28^ We used SSO transfection with LF2000 as transfection control and the previously published endosomal escape enhancer CMP05 (here, EEE2) as positive control.^15^ Results showed efficient splice-switching using two of the newly generated EEEs (EEE4 and EEE8). Levels of luciferase expression for both EEEs were similar to that of LF2000 and significantly higher than with EEE2, at 24 h EEE treatment using (2 μM) EEE in SSO pre-incubated cells (Figure 1B; Table S3). Several other EEEs enhanced splice-switching around 20-fold when compared with oligonucleotide alone; however, they did not perform better than EEE2 and were excluded from further studies (Figure 1B; Tables S2 and S3).

To better compare the two selected candidates, EEE4 and EEE8, a dose-response experiment was performed using half of the previously tested SSO concentration (1 μM). EEE4 was significantly more potent than EEE8 and efficiently enhanced SSO activity at concentrations between 1.5 and 3.5 μM, while EEE8 required an at least three times higher dose (4.5 μM) to show splice-switching above 10-fold when normalized to SSO only (Figure 1C, left). Dose-dependent cell toxicity was also assessed by measuring WST-1 cleavage, which occurs only in metabolically active cells. As expected, cellular viability was compromised upon treatment with high doses of EEEs. However, at efficient concentration (around 2 μM), EEE4 displayed low cellular toxicity (Figure 1C, right).

We further studied endosomal leakage by quantifying GAL9 recruitment upon EEE dose escalation, from 0.31 to 10 μM. For this purpose, we used cell lines carrying an mCherry-GAL9-reporter, where endosomal rupture could be quantified by measuring GAL9 clustering as a puncta event representative of endosomal remodeling. Results from the GAL9 assay revealed clear puncta formation from 1.25 μM for EEE4 and EEE8, whereas DMSO-treated cells presented a homogeneous cytoplasmic GAL9 pattern (Figure 1D, left). A radar chart comparing GAL9-recruitment in eight different cell lines upon EEE4 and EEE8 treatment allowed us to visualize that EEE4 has broader tissue action and higher levels of GAL9 puncta formation at lower concentrations, indicating more potent endosomal leakage than EEE8 (Figure 1D, right).

### Characterization of EEE4 as an endosomal escape enhancer

Further studies evaluating acute toxicity upon EEE4 treatment were performed by measuring extracellular lactate dehydrogenase (LDH) release as indicator of possible plasma membrane damage.

**Figure 2:**
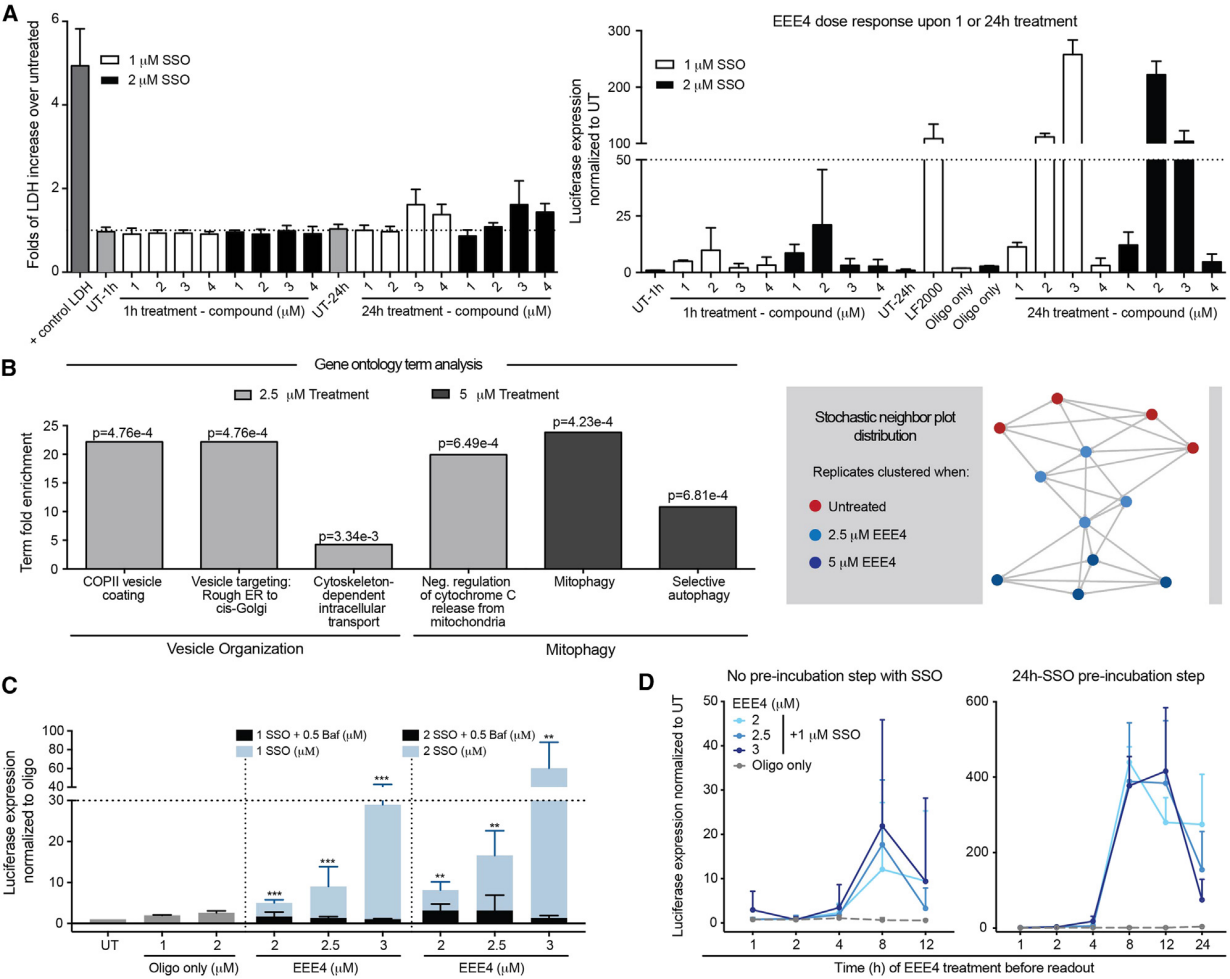
Functional studies of EEE4 as an endosomal escape enhancer (A) Efficient EEE4 concentration showed no to mild-acute cell toxicity upon 1- to 24-h treatment. For luciferase expression analysis, SSO-pretreated cells were EEE treated and then rinsed, 0.1% Triton X-100 lysed, and luciferase intensity measured. Conditioned media was collected 1 or 24 h after treatment initiated for lactate dehydrogenase cell toxicity assay and performed according to the manufacturer’s instructions. (B) (Bar plots) Selected Gene Ontology term analysis on bulk RNA sequencing data, fold enrichments, and *p* values for genes upregulated by 2.5-μM (light gray) or 5-μM (dark gray) treatments. (Plot) Force directed t-stochastic neighbor embedding (t-SNE) nearest neighbor plot showing connections between each experiment and its four nearest neighbors. (C) EEE4 potency is dependent on endosomal acidification. H^+^ATPase inhibition was performed by EEE4 + bafilomycin (0.5 μM) co-incubation. (D) The 24-h SSO pre-incubation enhanced endosomal escape EEE4 activity. Cells were either SSO + EEE4 cotreated or 24 h SSO pre-incubated and then EEE4 treated, followed by rinse, lysis, and luciferase readout at five different time points after EEE4 treatment. (A, C, and D) Bars indicate mean and SD. Significance was calculated for single variable using the Mann-Whitney *U* test (∗*p* < 0.05; ∗∗*p* < 0.005; ∗∗∗*p* < 0.0005).

No acute toxicity was observed for the tested concentrations, as LDH levels measured at 1-h treatment were similar to untreated controls; however, at 24-h treatment, a slight increase in LDH release was observed upon treatment with EEE4 at ≥3 μM concentrations. Luciferase expression and WST-1 cleavage showed that cells treated with EEE4 for 24 h presented enhanced SSO activity and mild to no cytotoxic effect at 2–3 μM concentrations (Figure 2A).

As with the previous generation of EEEs, we hypothesized that EEE4 interferes with the SSO trafficking and induces oligonucleotide escape by endosomal membrane rupture. We performed a battery of tests to further understand its mechanism of action.

To study the global impact of EEE4, we performed Gene Ontology (GO) analysis on bulk RNA sequencing data from treated cells. Selected GO term analysis showed that EEE4 treatment induces differential gene expression associated with vesicle organization or mitophagy. As such, cells treated with 2.5 μM EEE4 concentration have an enrichment of markers associated with cell organization processes, such as vesicle formation, targeting, and transportation. In contrast, high EEE4 concentration provided insight into the mechanisms of mediated toxicity (Figure 2B, left). t-Stochastic neighbor embedding (t-SNE) confirmed the association between the different EEE4 treatment concentrations by revealing clustering of RNA-sequenced sample replicates (Figure 2B, right).

Next, we set out to determine the mode of action of EEE4 on endosomal escape. We have previously shown with EEE2 that acidification of endosomes is critical to the activity of the EEE. Similarly, here, using EEE4 co-treatment with the H^+^ATPase inhibitor bafilomycin A1, we observed significantly reduced SSO-mediated splice-switching. These results strongly suggest a proton sponge type of endosomal escape mechanism for EEE4 (Figure 2C). Based on previous findings using EEE2, we hypothesized that simultaneous SSO + EEE4 uptake is needed to induce efficient splice-switching. To evaluate this hypothesis, we performed a parallel experiment using EEE4 dose response with either SSO co-treatment or 24-h SSO pre-incubation step. Luciferase readout was performed at five different time points after EEE4 addition, and splice-switching was observed under both experimental conditions. Surprisingly, splice-switching in the SSO pre-incubation group was 20 times higher than that observed for EEE4-SSO co-treatment (Figure 2D). Finally, to ascertain that EEE4 indeed acts by enhancing the endosomal release of ASOs and not merely by increasing cellular uptake, a cellular uptake experiment was performed using AF568-labeled SSO. As expected, we did not observe any enhanced cellular uptake of SSOs in the presence of EEE4, as measured by flow cytometry (Figure S2). These results confirm that EEE4 enhances endosomal escape while having a negligible impact on cellular uptake.

### Gem-dimethyl substitution on EEE4 piperidine led to lower toxicity and retained endosomal escape activity

**Figure 3:**
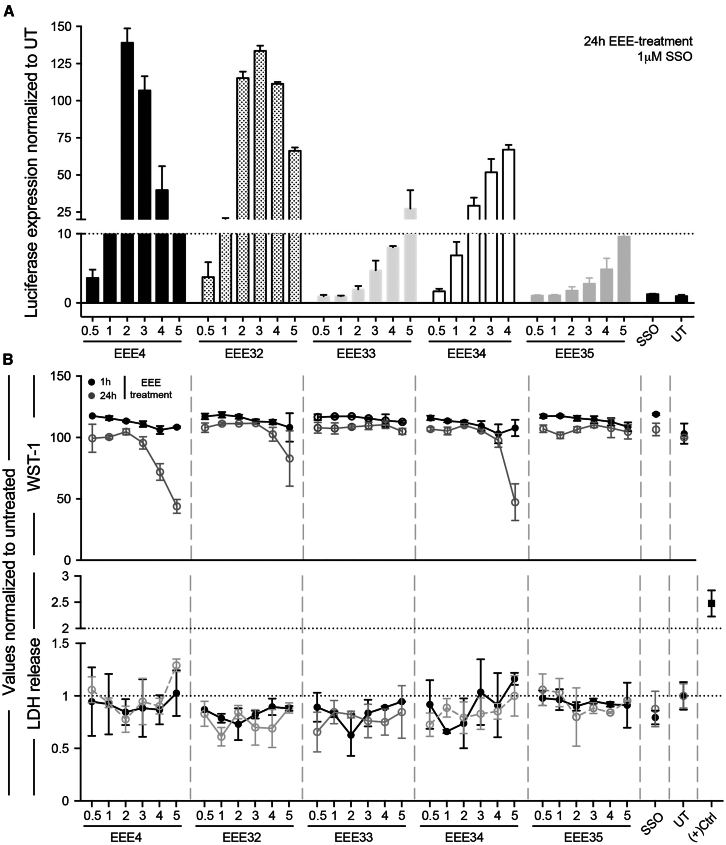
EEE4 piperidine chemical substitutions are well tolerated and have positive impact on cell viability (A) HeLa_Luc705 cells were SSO pretreated, followed by either 1- or 24-h EEE treatment, rinse, lysis and luciferase measurements. (B) Conditioned media was collected 1 or 24 h after treatment initiated for lactate dehydrogenase cell toxicity assay and performed according to the manufacturer’s instructions. For tetrazolium salt WST-1 assay, the cytotoxic effect was measured 1 or 24 h after treatment initiation, considering WST-1 prior incubation time (4 h) according to the manufacturer’s instructions. Bars indicate mean and SD.

We further investigated the impact of introducing modifications on ring D since it tolerates more chemical diversity than other rings (Figure 1A). The pyrazole connection site on the piperidine (position 3 or 4) proved to have no impact on the activity of the EEEs, and introducing additional alkyl substituents did not cause a loss of potency (Tables S1–S3). Substitution with gem-dimethyl on position 2 led to lower toxicity (as shown in the LDH release and WST-1 assays) while retaining activity (Figure 3). We hypothesize that this increased tolerability might be associated with the lower nucleophilicity of the sterically hindered piperidine nitrogen, thus preventing potential undesired interactions in cells. In addition, alkylation of the nitrogen atom preserves its Brønsted basicity, which appears to be a critical parameter for EEE potency, as further shown by the total loss of activity observed upon acetylation of this atom (Tables S1–S3). Thus, functional screening using the luciferase splice-switching assay as well as cellular toxicity indicates that two new EEEs are promising, EEE32 and EEE34 (Figure 3). However, none of these EEEs was superior to EEE4, and only EEE32 showed similar potency (Figure 3A).

### EEE32 as a multimodal oligonucleotide therapeutics enhancer

**Figure 4:**
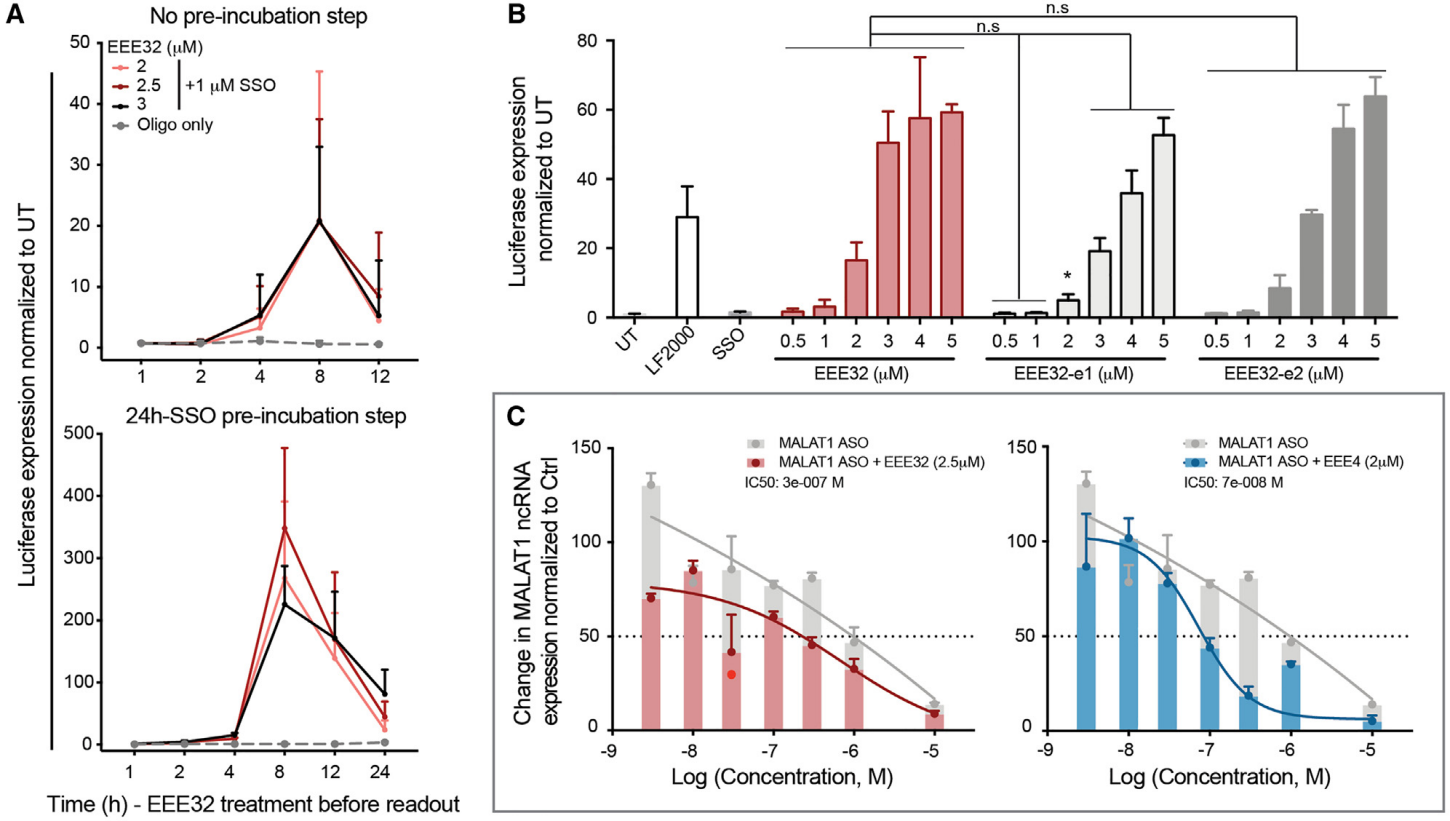
EEEs as endosomal escape enhancer for SSO and gapmer (A) Similar to EEE4, 24-h SSO pre-incubation enhanced EEE32-induced endosomal escape activity. Cells were either cotreated or 24-h SSO pre-incubated and then EEE4 treated, followed by rinse, lysis, and luciferase readout at indicated time points. (B) No changes in luciferase expression were observed upon SSO delivery with EEE32 enantiomers. HeLa_Luc705 cells were treated and luciferase expression measured as previously described. (C) Cells were seeded, 24 h after ASO + EEE treatment, followed by rinse and sample collection at 24 h. Dose response in moles for MALAT1 ASO (1e−5, 3e−5, …, 3e−9, 1e−9). Bars indicate mean with SD. Significance for multiple variables comparison was calculated using multiple t test Sidak-Bonferroni correction method (α = 0.05) (n.s, not significant; ∗*p* < 0.05).

Based on the encouraging dose-response results with EEE4, we next performed a dose-response experiment with EEE32, including 1 μM SSO co-treatment or a 24-h SSO pre-incubation step. The luciferase readout for EEE32 almost replicated the results observed for EEE4, this time with a small difference in potency at the 24-h SSO pre-incubation step, where SSO activity was around 17 times higher than co-treatment (Figure 4A). To further understand whether structural molecule interactions are involved in the mechanism of action of EEE32, EEE32 enantiomers were produced. Results from the splice-switching analysis showed no differences between the generated structures when compared with EEE32, meaning that a structural interaction is less likely to occur (Figure 4B).

To fully realize the potential of these new EEEs to enhance the activity of oligonucleotides, we next expanded our testing to include a gapmer and siRNAs.

The tested gapmer was directed against the long non-coding RNA (lncRNA) MALAT1. The reason for choosing this target is that it is highly expressed in most cell types, and there are established efficient sequences used.^29^ Transcript knockdown was measured by quantifying MALAT1 lncRNA transcripts using quantitative polymerase chain reaction (qPCR); acidic ribosomal phosphoprotein PO (36B4) was used as housekeeping control. N2a cells were treated with a decreasing amount of MALAT1 gapmer and EEE treated for 24 h using the optimal identified concentration, 2 μM for EEE4 and 2.5 μM for EEE32. We observed that EEEs enhanced MALAT1 lncRNA knockdown and, interestingly, EEE4 (half-maximal inhibitory concentration [IC_50_]: 7.3 nM) was more efficient than EEE32 (IC_50_: 38.2 nM) (Figure 4C).

**Figure 5:**
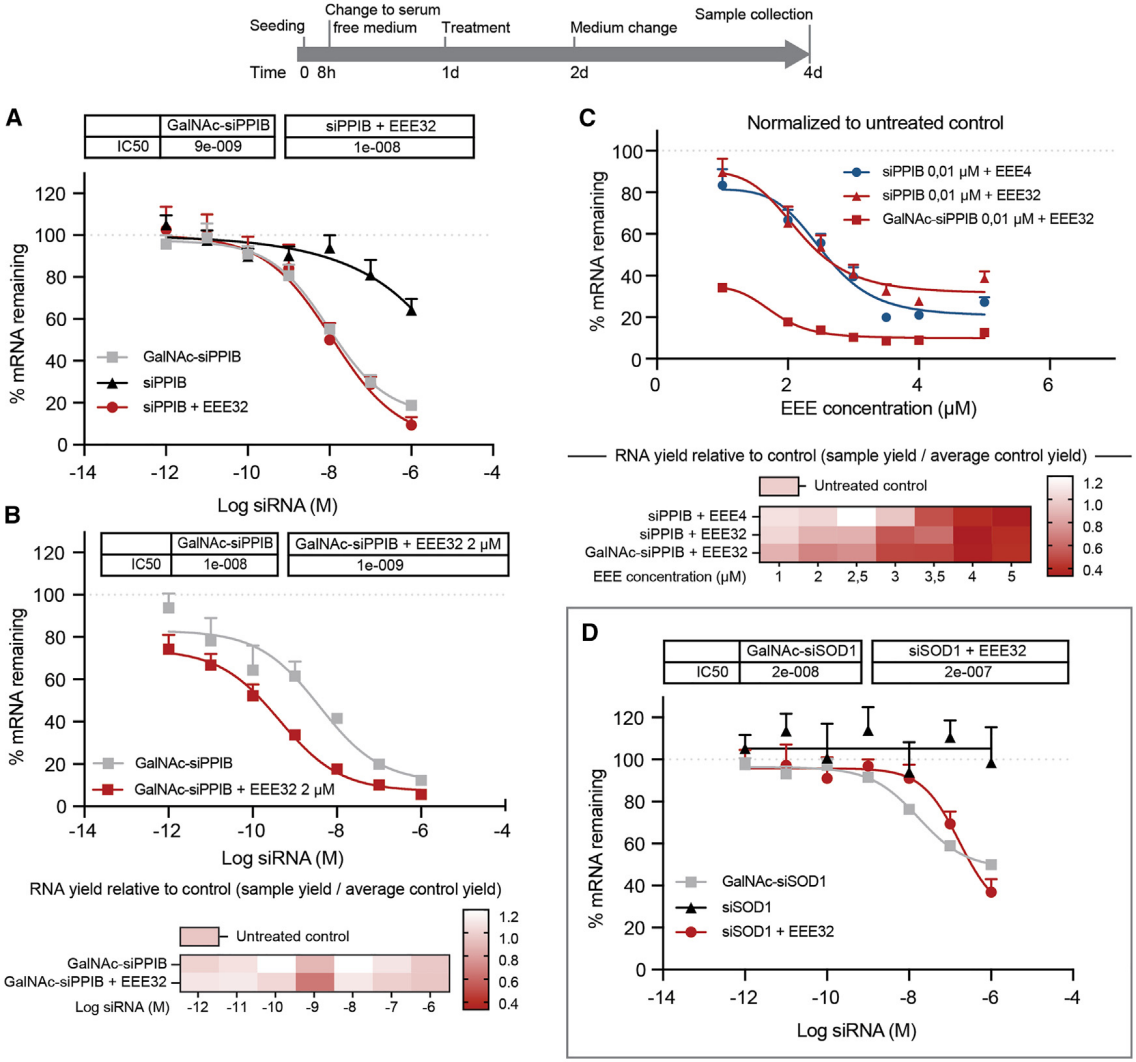
EEEs as endosomal escape enhancer for siRNA therapeutics (A–D) Cells were seeded 24 h after siRNA + EEE treatment, followed by rinse and sample collection at 48 h. (A, B, and D) Dose response for siRNA (1e−6, 1e−7, …, 1e−12). (C) Dose response for EEE 1, 2, 2.5, 3, 3.5, 4, and 5 μM. The heatmap shows RNA yield average of three replicates, calculated as (measured sample yield/measured control yield). Bars indicate mean with SD. Significance for multiple variables comparison was calculated using multiple t test Sidak-Bonferroni correction method (α = 0.05) (n.s, not significant; ∗*p* < 0.05).

Studies of siRNA knockdown therapeutics were performed in primary human hepatocytes using EEE32-mediated enhancement. We aimed to target peptidylpropyl isomerase B (PPIB) mRNA by using siRNA (siPPIB) either alone or together with the EEE32 and comparing them with GalNAc-conjugated siRNA (GalNAc-siPPIB), which is considered a potent human liver uptake enhancer.^30^ We did not observe any cell death using these treatments or combinations (morphology assessment under microscope and monitoring the Ct values of the housekeeping gene). Knockdown results showed that siPPIB function was enhanced when used together with EEE32 (IC_50_: 10 nM), while siPPIB alone did not reach the theoretically calculated IC_50_ at the tested concentrations from 1e−6 to 1 μM (estimated IC_50_: 7 μM) (Figures 5A and 5B). No differences were found when comparing IC_50_ from siPPIB + EEE32 (10 nM) with GalNAc-siPPIB (9 nM) (Figure 5A). However, the use of EEE32 at a 2-μM concentration enhanced GalNAc-siPPIB activity, improving the knockdown capacity by approximately 10-fold (Figure 5B, top). Additionally, no significant differences indicating compromised cell viability were observed when measuring systematic decreases in RNA yield from both groups (Figure 5B, bottom).

Furthermore, by using an EEE32/EEE4 dose titration, we performed a head-to-head comparison to assess the efficacy of the EEEs at a suboptimal siPPIB concentration (0.01 μM). Interestingly, the results showed efficient and similar or almost identical PPIB mRNA knockdown for both EEEs (Figure 5C, top). No significant differences indicating compromised cell viability were observed, and, as expected for these EEEs, concentrations ≥3 μM systematically decreased the measured RNA yield (Figure 5C, bottom). Conjugated GalNAc-siPPIB was also tested using an EEE32 titration, and the results confirmed the greater knockdown effect previously observed, opening a promising opportunity where conjugated siRNA can potentially be used alongside EEEs (Figure 5C).

Knockdown results from a second siRNA target, superoxide dismutase 1 (SOD1), confirmed our finding, and SOD1 mRNA expression was reduced by >60% by siSOD1 + EEE32, while duplex SOD1 alone was inefficient. GalNAc-siSOD1 treatment resulted in a maximum 50% knockdown of the SOD1 gene (Figure 5D).

## Discussion

At the time of writing this study, 22 oligonucleotide therapies were already approved worldwide for disorders that include specific targeting in the central nervous system, skeletal muscle, eye, and liver. Despite this evident success, oligonucleotide therapeutics still suffer from very inefficient endosomal escape.^12^^,^^13^^,^^31^ In this study, we present a set of SAR-explored EEE compounds that enhanced the activity of co-delivered ASOs with mild to negligible associated cytotoxicity. The SAR evaluation showed that EWG significantly boosted the potency of this particular type of EEEs.

Our data assessing the activity of the delivered SSO revealed splice-switching enhancement above 100-fold in using 2–2.5 μM of the most potent compounds, EEE4 and EE32. The best-enhanced activity observed was 400-fold higher than SSO alone, when using SSO pre-incubation and readout at 8 h treatment. Published results from previous EEE generations did not show this level of SSO activity enhancement, and the most efficient non-toxic concentration observed was higher than 2.5 μM.^15^ In previous publications, we used the same SSO non-covalently formulated with CPPs and showed an up to 125-fold SSO activity enhancement. Although this study reports experiments on EEE-mediated delivery of SSOs with only a phosphorothioate backbone, we see greater naked SSO activity enhancement than when using transfection reagents based on CPPs or LF2000.^32^

The endosomal escape properties of the generated EEE were assessed by using the GAL9 assay, a previously shown highly sensitive method used to characterize small molecules with membrane-disruptive properties.^33^ Our data revealed efficient GAL9 clustering in a wide spectrum of cells, suggesting membrane rupture as a cell-type-independent mechanism of action, while as reported for other modalities such as LNP siRNA, this seems to be restricted to the cell origin.^34^ Next, we sought to identify the mode of action of EEEs by assessing SSO activity upon inhibition of the endosome acidification mechanism using bafilomycin A1 co-treatment or by the generation of EEE enantiomers to identify possible structural molecule interactions. We were able to observe that bafilomycin A1 impaired EEE-SSO activity enhancement, and no differences in SSO activity were observed between the generated enantiomers. We hypothesize that simultaneous SSO + EEE4 uptake is needed to induce efficient splice-switching. However, results indicated that this is not fully required, as the splice-switching in the SSO pre-incubation group was 20 times higher than the EEE4-SSO co-treatment group. This suggests that the EEE molecule can penetrate the endosome lumen after cargo uptake and that cargo accumulation over time will ultimately be beneficial for the system. Additionally, AF568 -abeled SSO uptake experiments confirmed that EEE enhancement is independent of cellular uptake. Therefore, we conclude that the EEE mode of action derived from a proton sponge effect and that it is not involved in molecule-molecule interaction or does not affect gross oligonucleotide uptake.

The GO analysis on bulk RNA sequencing data allowed us to further understand the global effect of EEE4 on intracellular cargo management. We hypothesized that upon EEE4-induced endosomal rupture, cells activated a compensatory mechanism associated with vesicle reorganization and cargo transportation, shown by the enrichment of rough endoplasmic reticulum to Golgi transportation markers and cargo-selection/cargo-collection coat protein complex-II machinery.^35^^,^^36^^,^^37^ Interestingly, we found that 5-μM EEE4-concentration is associated with the upregulation of genes regulating mitophagy. This could be expected since mitochondria also have their own proton gradient, and accumulation of the EEE in mitochondria may cause mitochondrial disruption specifically in addition to endosomes.^38^^,^^39^^,^^40^ However, the most efficient EEE4 concentration (2.5 μM) did not induce mitophagy enrichment, and 1- to 24-h treatment induced no to mild cell toxicity, the latter being further improved by adding a gem-dimethyl group on position 2 of EEE32 without sacrificing oligonucleotide activity enhancement. This was evaluated by using luciferase expression, LDH leakage, and WST-1 cleavage.

Other oligonucleotide modalities of current clinical interest are siRNAs. In the last 5 years, 6 ASO and 5 siRNA drugs have been approved,^12^ and a simple search at ClinicalTrials.gov allowed us to identify 44/161 ASO and 51/140 siRNA active clinical studies, demonstrating the growing interest in these modalities. In this study, we showed enhanced activity of the very-well-established naked MALAT 1 gapmer and potency upon EEE treatment (IC_50_ for EEE4: 7.3 nM and EEE32: 68.6 nM), similar to what was observed in previous reports (IC_50_: 19 nM) upon covalent conjugation of MALAT1 gapmer with an estrogen-engineered GLP1R ligand.^29^ Results from siRNA targeting correlates with MALAT1 gapmer data, where EEE32 enhanced PPIB and SOD1 siRNA activity to an extent similar to that of GalNAc-conjugated PPIB and SOD1. Here, we are also demonstrating the potential of EEE32 to enhance GalNAc-conjugated siRNA without compromising the cell viability. However, deeper toxicity studies are required, and other conjugates targeting alternative receptors that induce active uptake could be explored to confirm these findings.^41^ Additionally, the EEE32-EEE4 head-to-head comparison demonstrated the potential of both EEEs to be used as siRNA enhancers. Other studies using compounds with a similar mode of action like chloroquine showed enhanced IC_50_ of chol-siGFP from 289 to 17 nM.^33^

In conclusion, here, we are disclosing novel SAR-generated EEEs that efficiently enhance the activity of multiple oligonucleotide therapeutics, with mild to no cytotoxicity and at lower concentrations in comparison to previous generations. We characterized these EEEs and identified the endosomal leakage as the main mechanism of action, modified the structural composition of the lead EEE, and reduced its induced cell toxicity while retaining the enhancing activity. Thus, we believe the set of EEEs disclosed here are molecules with a great potential to improve the therapeutic window of oligonucleotide-based therapies, with a reduced margin of toxicity that allows future preclinical applications. However, greater challenges must be overcome for successful *in vivo* translation,^42^ such as uneven cargo/EEE biodistribution, which could promote inefficient rupture of cargo-free endosomes, cellular and systemic toxicity, or bioaccumulation caused by the inability to cross natural barriers. We consider EEE4 and EEE32 a significant step forward, as we have reached the *in vitro* “sweet spot” that overcomes the fine line between optimal endosomal rupture and acceptable cellular stress. We demonstrate a synergistic effect between EEE and the already-efficient liver uptake enhancer GalNAc, opening the potential for GalNAc-siRNA-EEE conjugates.

## Materials and methods

### EEE synthesis

All synthesis details and analysis data are provided in the supplemental information.

### GAL9 recruitment assay

Experiments assessing GAL9 localization as an indicator for endosomal remodeling were performed as previously described^15^ using 8× stablemCherry-GAL9 expressing cell lines, SHSY5Y, U2OS, 16HBE, NCI-H358, HuH7, HepG2, HEK293, and HeLa. For mCherry-GAL9 recruitment, 3,000 or 6,500 cells/well (depending upon cell line) were seeded into 384-well CellCarrier Ultra plates 24 h prior to treatment (PerkinElmer, catalog no. 6007558). An Echo 655T acoustic dispenser (Labcyte) was used for EEE dose-response preparation by transferring to the cell plate. At assay endpoints, cells were PBS washed, 4% paraformaldehyde fixed at room temperature in degrees Celsius (VWR, catalog no. 9713.1000), and nuclei stained with PBS +1 μg/mL Hoechst 33342 (Thermo Fisher Scientific, catalog no. H21492).

Imaging was performed using a spinning-disk confocal microscope (Yokogawa: CV8000) with a ×20w objective (numerical aperture 1.0). Cells and mCherry-GAL9 structure identification and quantification were done using Columbus image-analysis software (PerkinElmer version 2.9.0). Data processing was done using Spotfire (Tibco version 11.4) and Prism (GraphPad version 9.1.0). Image panels were assembled using the FigureJ plugin for FIJI.^43^ All mCherry-GAL9 experiments were performed three times (*N* = 3) with duplicated wells per treatment (*n* = 2).

### Oligonucleotide therapeutics functional delivery assessment

#### Splice-switching oligonucleotide

For the assessment of functional SSO therapeutics, a HeLa_Luc705 cell line was grown in DMEM media supplemented with 10% fetal bovine serum (FBS). For most treatments, 10,000 cells/well were seeded in a 96-well plate, 24 h pre-incubated with 1- to 2-μM oligonucleotide, and then treated at the described EEE concentration. At 24 h post-treatment, cells were PBS rinsed and collected for further analysis (treatments and incubation times changed accordingly to the experiment design). Luciferase readout was performed as previously described,^15^ using a GloMax 96-well plate microplate luminometer (Promega) immediately after adding 30 μL auto-injected luciferin substrate (Promega Firefly Luciferase Assay System; Thermo Scientific, catalog no. 16174) in a white-walled 96-well plate containing 30 μL 0.1% Triton X-100 (Sigma-Aldrich) cell lysed. Photon exposure time and delay were set to 10 and 2 s after injection. LF2000 was used as the transfection reagent control (Thermo Scientific, catalog no. 11668019). For EEE experiments using 1 or 2 μM SSOs, the control was prepared using lipofection with 100–200 nM SSO; this is to avoid complex induced cell toxicity.

705-SSO was purchased from and synthesized by Integrated DNA Technologies. The 705-SSO sequence was previously published, containing 2′*-O-*methylated bases and only phosphorothioate-saturated backbone linkages were included.^15^ The 705-SSO was high-performance liquid chromatography purified, shipped, and stored in IDTE buffer (10 mM Tris, 0.1 mM EDTA).

#### Antisense oligonucleotide

The N2a cell line was grown and kept in 10% FBS-supplemented DMEM. For all treatments, 20,000 cells/well were seeded in a 96-well plate and ASO + EEE treated for 24 h before harvest and RNA isolation. RNA was extracted using the Maxwell RSC simplyRNA Cells Kit (Promega, catalog no. AS1390) and quantified using the Quant-iT RNA Assay Kit, Broad Range (Invitrogen, catalog no. Q10213) following the manufacturer’s instructions. The High-Capacity cDNA Reverse Transcription Kit (Applied Biosystems, catalog no. 4368814) was used to generate cDNA and TaqMan Fast Advanced Master Mix (Applied Biosystems, catalog no. 4444556) for qPCR reaction.

The following ASO primer/probe sequences were used: MALAT1 ASO sequence G∗C∗A∗T∗T∗mC∗T∗A∗A∗T∗A∗G∗mC∗A∗G∗C (∗phosphorothioate bond; m, 2′-*O*-methyl base; underline, locked nucleic acid); TaqMan assay probes designed for *Mus musculus*, ID: Mm01227912_s1; sequences are of a proprietary nature (Thermo Fisher TaqMan gene expression assay [FAM dye label], catalog no. 4351370) probe is FAM labeled and minor groove binder quencher; 36B4 forward: 5′-GAGGAATCAGATGAGGATATGGGA-3′, reverse: 5′-AAGCAGGCTGACTTGGTTGC-3′ and probe 5′-TCGGTCTCTTCGACTAATCCCGCCAA-3′.

### siRNA

Human primary hepatocytes obtained from a commercial supplier (QNT lot, BioIVT) were cultured in rat tail collagen I-coated 96-well plates (Corning, catalog no. 354407) using William’s E Medium (A1217601,Thermo Fisher) supplemented with thawing and plating supplements (Thermo Fisher, catalog no. CM3000) (day 0). The hepatocytes were maintained in William’s E Medium supplemented with hepatocyte maintenance supplements (Thermo Fisher, catalog no. CM4000) and 5C supplements (DAPT [catalog no. D5942], SB431542 [catalog no. 616461], forskolin [catalog no. F3917], IWP2 [catalog no. I0536], and LDN193189 [catalog no. SML0559], all from Merck), and cultured at 37°C in a humidified incubator with 5% CO_2_.

Following plating (day 1), the cells were treated with PPIB and SOD1 duplex or GalNAc-conjugated siRNAs and PBS in maintenance media. The EEE (2 μM) was cotreated with duplex siRNAs. After 24 h of treatment, the media was changed, and the cells were harvested on day 4 (72 h from the start of treatment) for qPCR analysis. Total RNA was extracted using a standard protocol (RNeasy Micro Kit, Qiagen, catalog no. 74004), and cDNA synthesis (High-Capacity cDNA Reverse Transcription Kit, Thermo Fisher, catalog no. 4368814), and qPCR was conducted using TaqMan primers (PPIB [Hs00168719_m1], SOD1 [Hs00533490_m1], and GAPDH [Hs04420632_g1]) and TaqMan Fast Advanced Master Mix (Thermo Fisher, catalog no. 4444964).

### SOD1 siRNA sequences

Guide: 5′-UUCAUUUCCACCUUUGCCCAAGU-3′
Hierarchical Editing Language for Macromolecules (HELM): 5′-{[mR](U)[sP].[fR](U)[sP].[mR](C)P.[mR](A)P.[mR](U)P.[fR](U)P.[mR](U)P.[fR](C)P.[fR](C)P.[mR](A)P.[mR](C)P.[mR](C)P.[mR](U)P.[fR](U)P.[mR](U)P.[fR](G)P.[mR](C)P.[mR](C)P.[mR](C)P.[mR](A)P.[mR](A)[sP].[mR](G)[sP].[mR](U)}-3′
Passenger: 5′-UUGGGCAAAGGUGGAAAUGAA-3′
HELM: 5′-{[mR](U)[sP].[mR](U)[sP].[mR](G)P.[mR](G)P.[mR](G)P.[mR](C)P.[fR](A)P.[mR](A)P.[fR](A)P.[fR](G)P.[fR](G)P.[mR](U)P.[mR](G)P.[mR](G)P.[mR](A)P.[mR](A)P.[mR](A)P.[mR](U)P.[mR](G)P.[mR](A)P.[mR](A)}-3′

### PPIB siRNA sequences

Guide: 5′-UCACGAUGGAAUUUGCUGUU-3′
HELM: 5′-{[mR](U)[sP].[fR](C)[sP].[mR](A)P.[fR](C)P.[mR](G)P.[fR](A)P.[mR](U)P.[fR](G)P.[mR](G)P.[fR](A)P.[mR](A)P.[fR](U)P.[mR](U)P.[fR](U)P.[mR](G)P.[fR](C)P.[mR](U)P.[fR](G)[sP].[mR](U)[sP].[fR](U)}-3′
Passenger: 5′-CAGCAAAUUCCAUCGUGA-3′
HELM: 5′-{[mR](C)[sP].[fR](A)[sP].[mR](G)P.[fR](C)P.[mR](A)P.[fR](A)P.[mR](A)P.[fR](U)P.[mR](U)P.[fR](C)P.[mR](C)P.[fR](A)P.[mR](U)P.[fR](C)P.[mR](G)P.[fR](U)[sP].[mR](G)[sP].[fR](A)}-3′

### Cell toxicity assays

For measuring cell membrane damage, extracellular LDH was quantified in conditioned media collected 1 or 24 h after initiation of treatment. LDH quantification was performed following the manufacturer’s instructions (Thermo Fisher Scientific, catalog no. C20300), 0.1% Triton X-100 cell lysed was used as the positive control, and values were normalized to untreated control.

For measuring the EEE cytotoxic effect, formazan formation was quantified upon tetrazolium salt WST-1 cleavage 1 or 24 h after initiation of treatment. As recommended by the manufacturer (Roche, catalog no. 5015944001), a 4-h WST-1 pre-incubation step was included before formazan quantification. Absorbance was measured between 420 and 480 nm, background signal was considered in the calculation, and values were normalized to untreated control.

### GO analysis

To judge the global effects of EEE on cells, HeLa cells were treated with 2.5 or 5 μM EEE4 for 24 h. Cells were harvested, cDNA from bulk RNA was prepared for sequencing using the Smart-seq2 protocol, as in Hagey et al.,^44^ and single-end 50-bp sequencing of samples was carried out on an Illumina HiSeq3000 machine. Reads per million per kilobase gene were calculated, and the most variable genes were used to map and cluster samples using a t-SNE-nearest neighbor method in R. Differential expression between quadruplicate samples was performed using the Deseq2 package in R, and genes upregulated in the treatment groups with padj < 0.05 were used as input into the Panther GO database.

## Data availability

Researchers requesting original data should contact the corresponding authors H.Y.E., A.D., or S.EL.A.

## Acknowledgments

H.Y.E. would like to acknowledge Minciencias - COLCIENCIAS, scholarship program 756 (2016). H.Y.E. and S.EL.A. thank Fredrik Höök and the Swedish Foundation for Strategic Research FoRmulaEx consortium (SM19-0007) for financial support. S.EL.A. would like to acknowledge the 10.13039/501100000781European Research Council under the European Union’s Horizon 2020 research and innovation programme (DELIVER, grant no. 101001374), ⁠the European Union’s Horizon 2020 research and innovation programme (EXPERT, grant no. 825828), the 10.13039/501100002794Swedish Cancer Society, the 10.13039/501100004359Swedish Research Council (4-258/2021), and the Novo Nordisk Foundation’s Distinguished Innovator Grant.

## Author contributions

H.Y.E., T.B., S.EL.A., and A.D. conceptualized, discussed, and developed the project. H.Y.E., S.R., and J.B. performed experiments aimed at the assessment of functional SSO and ASO delivery. H.Y.E., D.V.-R., and O.G. carried out the pre-incubation and uptake assessments. T.B., S.E.L.A., and A.D. carried out the SAR studies. D.K.B. and D. Hekman performed the siRNA delivery experiments. D. Hagey carried out the GO term and stochastic neighbor analysis. M.J.M. performed the GAL9 response assay. H.Y.E., T.B., M.J.M., S.A., S.E.L.A., and A.D. analyzed and interpreted results, participated in critical discussions, and provided funding and project direction. T.B. and M.J.M. wrote selected parts of the manuscript. H.Y.E., S.E.L.A., and A.D. wrote the manuscript.

## Declaration of interests

H.Y.E., O.G., S.A., J.B., S.E.L.A., and A.D. are listed as inventors on an international patent application entitled “Compositions and Methods for Delivering a Macromolecule to a Cell.” T.B., M.J.M., D.K.B., D. Hekman, S.A., and A.D. are employees of AstraZeneca.

## Supplemental information

Document S1. Figures S1 and S2, Tables S1–S3, and supplemental methods

Document S2. Article plus supplemental information

## References

[1] Juliano R.L. (2016). The delivery of therapeutic oligonucleotides. Nucleic Acids Res..

[2] Bennett C.F., Swayze E.E. (2010). RNA targeting therapeutics: Molecular mechanisms of antisense oligonucleotides as a therapeutic platform. Annu. Rev. Pharmacol. Toxicol..

[3] Havens M.A., Hastings M.L. (2016). Splice-switching antisense oligonucleotides as therapeutic drugs. Nucleic Acids Res..

[4] Neil E.E., Bisaccia E.K. (2019). Nusinersen: A novel antisense oligonucleotide for the treatment of spinal muscular atrophy. J. Pediatr. Pharmacol. Therapeut..

[5] Kim J., Hu C., Moufawad El Achkar C., Black L.E., Douville J., Larson A., Pendergast M.K., Goldkind S.F., Lee E.A., Kuniholm A. (2019). Patient-Customized Oligonucleotide Therapy for a Rare Genetic Disease. N. Engl. J. Med..

[6] Bauman J., Jearawiriyapaisarn N., Kole R. (2009). Therapeutic potential of splice-switching oligonucleotides. Oligonucleotides.

[7] Bestas B., McClorey G., Tedebark U., Moreno P.M., Roberts T.C., Hammond S.M., Smith C.E., Wood M.J., Andaloussi S.E. (2014). Design and application of bispecific splice-switching oligonucleotides. Nucleic Acid Therapeut..

[8] Wilton-Clark H., Yokota T. (2023). Recent Trends in Antisense Therapies for Duchenne Muscular Dystrophy. Pharmaceutics.

[9] Smith C.I.E., Zain R. (2019). Therapeutic oligonucleotides: State of the art. Annu. Rev. Pharmacol. Toxicol..

[10] Bennett C.F., Krainer A.R., Cleveland D.W. (2019). Antisense Oligonucleotide Therapies for Neurodegenerative Diseases. Annu. Rev. Neurosci..

[11] Gavrilov K., Saltzman W.M. (2012). Therapeutic siRNA: Principles, challenges, and strategies. Yale J. Biol. Med..

[12] Androsavich J.R. (2024). Frameworks for transformational breakthroughs in RNA-based medicines. Nat. Rev. Drug Discov..

[13] Gilleron J., Querbes W., Zeigerer A., Borodovsky A., Marsico G., Schubert U., Manygoats K., Seifert S., Andree C., Stöter M. (2013). Image-based analysis of lipid nanoparticle-mediated siRNA delivery, intracellular trafficking and endosomal escape. Nat. Biotechnol..

[14] Hedlund H., Du Rietz H., Johansson J.M., Eriksson H.C., Zedan W., Huang L., Wallin J., Wittrup A. (2023). Single-cell quantification and dose-response of cytosolic siRNA delivery. Nat. Commun..

[15] Bost J.P., Ojansivu M., Munson M.J., Wesén E., Gallud A., Gupta D., Gustafsson O., Saher O., Rädler J., Higgins S.G. (2022). Novel endosomolytic compounds enable highly potent delivery of antisense oligonucleotides. Commun. Biol..

[16] Maxfield F.R. (1982). Weak bases and ionophores rapidly and reversibly raise the ph of endocytic vesicles in cultured mouse fibroblasts. J. Cell Biol..

[17] Andaloussi S.E.L., Lehto T., Mäger I., Rosenthal-Aizman K., Oprea I.I., Simonson O.E., Sork H., Ezzat K., Copolovici D.M., Kurrikoff K. (2011). Design of a peptide-based vector, PepFect6, for efficient delivery of siRNA in cell culture and systemically in vivo. Nucleic Acids Res..

[18] Lehto T., Simonson O.E., Mäger I., Ezzat K., Sork H., Copolovici D.M., Viola J.R., Zaghloul E.M., Lundin P., Moreno P.M.D. (2011). A peptide-based vector for efficient gene transfer in vitro and in vivo. Mol. Ther..

[19] Pelt J., Busatto S., Ferrari M., Thompson E.A., Mody K., Wolfram J. (2018). Chloroquine and nanoparticle drug delivery: A promising combination. Pharmacol. Ther..

[20] Wang T., Larcher L.M., Ma L., Veedu R.N. (2018). Systematic screening of commonly used commercial transfection reagents towards efficient transfection of single-stranded oligonucleotides. Molecules.

[21] Bizot F., Fayssoil A., Gastaldi C., Irawan T., Phongsavanh X., Mansart A., Tensorer T., Brisebard E., Garcia L., Juliano R.L., Goyenvalle A. (2023). Oligonucleotide Enhancing Compound Increases Tricyclo-DNA Mediated Exon-Skipping Efficacy in the Mdx Mouse Model. Cells.

[22] Juliano R.L., Wang L., Tavares F., Brown E.G., James L., Ariyarathna Y., Ming X., Mao C., Suto M. (2018). Structure-activity relationships and cellular mechanism of action of small molecules that enhance the delivery of oligonucleotides. Nucleic Acids Res..

[23] Wang L., Ariyarathna Y., Ming X., Yang B., James L.I., Kreda S.M., Porter M., Janzen W., Juliano R.L. (2017). A Novel Family of Small Molecules that Enhance the Intracellular Delivery and Pharmacological Effectiveness of Antisense and Splice Switching Oligonucleotides. ACS Chem. Biol..

[24] Yang B., Ming X., Cao C., Laing B., Yuan A., Porter M.A., Hull-Ryde E.A., Maddry J., Suto M., Janzen W.P., Juliano R.L. (2015). High-throughput screening identifies small molecules that enhance the pharmacological effects of oligonucleotides. Nucleic Acids Res..

[25] Havens M.A., Duelli D.M., Hastings M.L. (2013). Targeting RNA splicing for disease therapy. Wiley Interdiscip. Rev. RNA.

[26] Dominski Z., Kole R. (1993). Restoration of correct splicing in thalassemic pre-mRNA by antisense oligonucleotides. Proc. Natl. Acad. Sci. USA.

[27] Kang S.H., Cho M.J., Kole R. (1998). Up-regulation of luciferase gene expression with antisense oligonucleotides: Implications and applications in functional assay development. Biochemistry.

[28] Rocha C.S.J., Lundin K.E., Behlke M.A., Zain R., El Andaloussi S., Smith C.I.E. (2016). Four Novel Splice-Switch Reporter Cell Lines: Distinct Impact of Oligonucleotide Chemistry and Delivery Vector on Biological Activity. Nucleic Acid Therapeut..

[29] Ämmälä C., Drury W.J., Knerr L., Ahlstedt I., Stillemark-Billton P., Wennberg-Huldt C., Andersson E.M., Valeur E., Jansson-Löfmark R., Janzén D. (2018). Targeted delivery of antisense oligonucleotides to pancreatic β-cells. Sci. Adv..

[30] Boianelli A., Aoki Y., Ivanov M., Dahlén A., Gennemark P. (2022). Cross-Species Translation of Biophase Half-Life and Potency of GalNAc-Conjugated siRNAs. Nucleic Acid Therapeut..

[31] Al Shaer D., Al Musaimi O., Albericio F., de la Torre B.G. (2024). 2023 FDA TIDES (Peptides and Oligonucleotides) Harvest. Pharmaceuticals.

[32] Bazaz S., Lehto T., Tops R., Gissberg O., Gupta D., Bestas B., Bost J., Wiklander O.P.B., Sork H., Zaghloul E.M. (2021). Novel orthogonally hydrocarbon-modified cell-penetrating peptide nanoparticles mediate efficient delivery of splice-switching antisense oligonucleotides in vitro and in vivo. Biomedicines.

[33] Du Rietz H., Hedlund H., Wilhelmson S., Nordenfelt P., Wittrup A. (2020). Imaging small molecule-induced endosomal escape of siRNA. Nat. Commun..

[34] Gilleron J., Paramasivam P., Zeigerer A., Querbes W., Marsico G., Andree C., Seifert S., Amaya P., Stöter M., Koteliansky V. (2015). Identification of siRNA delivery enhancers by a chemical library screen. Nucleic Acids Res..

[35] Gürkan C., Stagg S.M., LaPointe P., Balch W.E. (2006). The COPII cage: Unifying principles of vesicle coat assembly. Nat. Rev. Mol. Cell Biol..

[36] Tang V.T., Ginsburg D. (2023). Cargo selection in endoplasmic reticulum–to–Golgi transport and relevant diseases. J. Clin. Investig..

[37] Zhang Y., Srivastava V., Zhang B. (2023). Mammalian cargo receptors for endoplasmic reticulum-to-Golgi transport: mechanisms and interactions. Biochem. Soc. Trans..

[38] Picca A., Guerra F., Calvani R., Coelho-Júnior H.J., Landi F., Bucci C., Marzetti E. (2023). Mitochondrial-Derived Vesicles: The Good, the Bad, and the Ugly. Int. J. Mol. Sci..

[39] Pickles S., Vigié P., Youle R.J. (2018). Mitophagy and Quality Control Mechanisms in Mitochondrial Maintenance. Curr. Biol..

[40] König T., McBride H.M. (2024). Mitochondrial-derived vesicles in metabolism, disease, and aging. Cell Metab..

[41] Kohashi H., Nagata R., Tamenori Y., Amatani T., Ueda Y., Mori Y., Kasahara Y., Obika S., Shimojo M. (2024). A novel transient receptor potential C3/C6 selective activator induces the cellular uptake of antisense oligonucleotides. Nucleic Acids Res..

[42] Allen R., Yokota T. (2024). Endosomal Escape and Nuclear Localization: Critical Barriers for Therapeutic Nucleic Acids. Molecules.

[43] Mutterer J., Zinck E. (2013). Quick-and-clean article figures with FigureJ. J. Microsc..

[44] Hagey D.W., Kvedaraite E., Akber M., Görgens A., Javadi J., von Bahr Greenwood T., Björklund C., Åkefeldt S.O., Hannegård-Hamrin T., Arnell H. (2023). Myeloid cells from Langerhans cell histiocytosis patients exhibit increased vesicle trafficking and an altered secretome capable of activating NK cells. Haematologica.

