# Supplementary material for: Design and screening of novel endosomal escape compounds that enhance functional delivery of oligonucleotides in vitro
Source: Mol Ther Nucleic Acids. 2025 Mar 20;36(2):102522. doi: 10.1016/j.omtn.2025.102522 (PMC11999280; doi:10.1016/j.omtn.2025.102522)
Supplement: Document S1. Figures S1 and S2, Tables S1–S3, and supplemental methods [file mmc1.pdf]

OMTN, Volume *36*

Supplemental information

Design and screening of novel endosomal escape compounds that enhance functional delivery of oligonucleotides *in vitro*

H. Yesid Estupiñán, Tom Baladi, Samantha Roudi, Michael J. Munson, Jeremy Bost, Oskar Gustafsson, Daniel Velásquez-Ramírez, Deepak Kumar Bhatt, Daniel Hagey, Dennis Hekman, Shalini Andersson, Samir EL Andaloussi, and Anders Dahlén

Supplemental Material

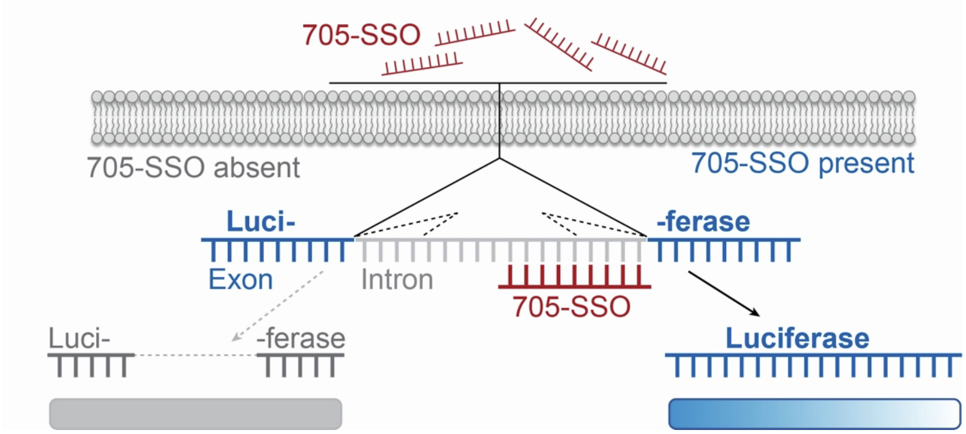

Figure S1. Schematic representation of the splice-correction strategy used in pLUC/705 reporter. Luc705 reporters carry a pLuc/705 splice-switching luciferase gene, interrupted by a mutated b-globin intron. This intron presents an aberrant 5' splice site, which activates a cryptic 3´ splice site that translates an aberrant luciferase protein. Hybridization of 705-splice-switching oligonucleotide (705-SSO) with the aberrant pre-mRNA, results in correct splicing and translated luciferase protein.

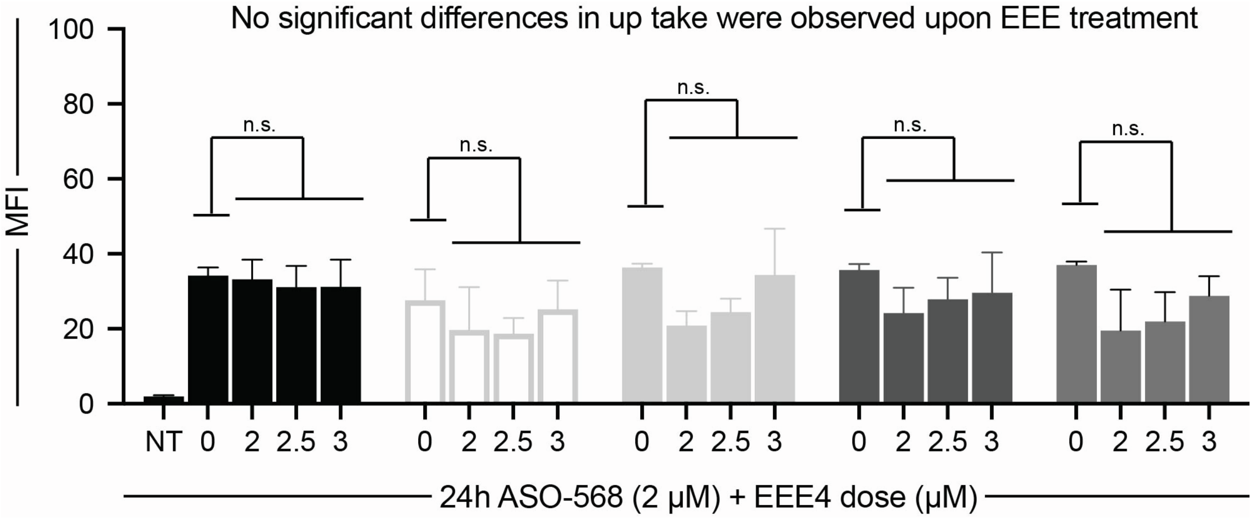

Figure S2. Uptake of ASO is not affected by EEE4 treatment. HeLa ells were either SSO + EEE4 cotreated or 24h-SSO pre-incubated and then EEE4 treated, followed by readout using flow cytometry at 0, 2, 4, 6 and 8h after EEE4-treatment. Significant was calculated, for single variable using Mann-Whitney U test (*P <0.05, **P <0.005 and ***P <0.0005).

Table S1
Structure activity relationship generated compounds with NMR spectra analysis

#	Chemical structure	MS (ESI+)	NMR	#	Chemical structure	MS (ESI+)	NMR
2	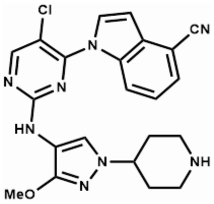	449.1	1H NMR: (400 MHz DMSO-d6): δ 8.44(s, 1H), 8.08 (s, 1H), 7.85 (d, J = 8.0 Hz, 1H), 7.73 (s, 1H), 7.61 (d, J = 8.0 Hz, 1H), 7.34 (t, J = 8.0 Hz ,1H), 3.90 (s, 1H), 3.77 (m, 3H), 2.96 (s, 2H), 1.84 (s, 2H), 1.67-1.60 (m, 2H).	6	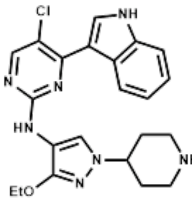	438.0	1H NMR (400 MHz DMSO-d6): δ ppm 11.82 (br s, 1 H), 8.48 (s, 1 H), 8.30 (s, 2 H), 7.70 (s, 1 H), 7.46 (d, J=7.54 Hz, 1 H), 7.18 (t, J=8.01 Hz, 1 H), 6.91 - 7.10 (m, 1 H), 4.15 (q, J=7.10 Hz, 2 H), 3.97 (dd, J=15.64,7.54 Hz, 1 H), 3.02 (d, J=18.84 Hz, 2 H), 2.55 - 2.67 (m, 2 H), 1.84 - 2.00 (m, 2 H), 1.56 - 1.84 (m, 2 H), 1.22 (t, J=6.97 Hz, 3 H)
3	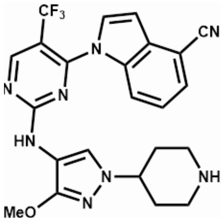	483.1	1H NMR: (400 MHz DMSO-d6): δ 8.84 (brs, 1H), 7.84 (d, J = 8.0 Hz, 1H), 7.72 (s, 1H), 7.56 (d, J = 7.2 Hz), 7.33 (t, J = 8 Hz, 1H), 3.91 (brs, 1H), 3.90 (s, 3H), 2.99 (s, 2H), 2.53 - 2.50 (m, 1H), 1.85 (s, 1H), 1.62 (brs, 1H).	7	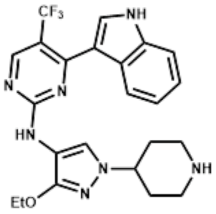	472.1	1H NMR: (400 MHz DMSO-d6): δ 9.03-8.88 (s, 1H), 8.60 (s, 1H), 8.52-7.96 (s, 1H), 7.85 (s, 1H), 7.74 (s, 1H), 7.45-7.43 (s, 1H), 7.10-6.96 (m, 2H), 4.30 (s, 2H), 3.85 (s, 1H), 3.02-2.99 (m, 2H), 2.66-2.55 (m, 2H), 1.88 (m, 2H), 1.70-1.67 (m, 2H), 1.26-1.17 (m, 3H).
4	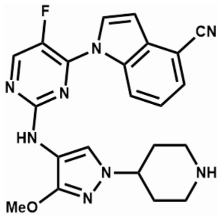	433.1	1H NMR: (400 MHz DMSO-d6): δ 8.81 (brs, 1H), 8.60 (d, J = 4.0 Hz, 1H), 8.08 (s, 1H), 7.73 (s, 1H), 7.32 (brs, 1H), 6.95 (d, J = 4.0 Hz, 1H), 3.99-3.94 (m, 1H), 3.79 (s, 1H), 3.01 (d, J = 8.0 Hz, 1H), 2.57-2.54 (m, 2H), 1.90-1.87 (m, 2H), 1.71-1.68 (m, 2H).	8	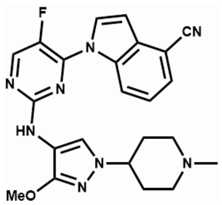	447.2	1H NMR (500 MHz, DMF-d7): δ 1.94 – 2.1 (m, 6H), 2.24 (s, 3H), 2.89 (d, J = 10.7 Hz, 2H), 3.89 (s, 3H), 3.96 (dd, J = 11.1, 5.4 Hz, 1H), 7.02 (d, J = 3.6 Hz, 1H), 7.44 (s, 1H), 7.79 (d, J = 7.4 Hz, 1H), 7.89 (s, 1H), 8.18 (dd, J = 3.6, 2.2 Hz, 1H), 8.63 (d, J = 4.3 Hz, 1H).
5	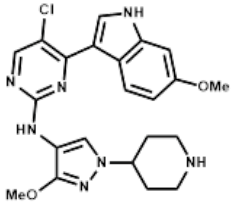	454.0	1H NMR (300 MHz, MeOD-d4): δ ppm 2.02 - 2.29 (m, 4 H) 3.01 - 3.16 (m, 2 H) 3.46 (dt, J=12.81, 3.58 Hz, 2 H) 3.75 (s, 3 H) 3.81 (s, 3 H) 4.10 - 4.31 (m, 1 H) 6.67 (d, J=8.48 Hz, 1 H) 6.89 (d, J=2.26 Hz, 1 H) 7.67 (s, 1 H) 8.12 (s, 2 H) 8.41 (s, 1 H)	9	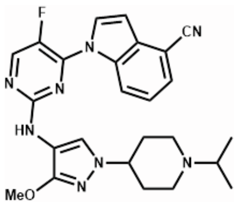	475.4	1H NMR (500 MHz, DMF-d7): δ 1.10 (s, 6H), 1.95 - 2.5 (m, 6H), 3.06 (bs, 2H), 3.89 (s, 3H), 4.03 (m, 1H), 7.03 (d, J = 3.6 Hz, 1H), 7.45 (t, J = 7.7 Hz, 1H), 7.78 (d, J = 7.4 Hz, 1H), 7.90 (s, 1H), 8.16 – 8.21 (m, 1H), 8.64 (d, J = 4.3 Hz, 1H).

#	Chemical structure	MS (ESI+)	NMR	#	Chemical structure	MS (ESI+)	NMR
10	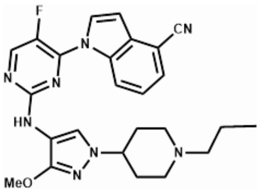	475.2	1H NMR (500 MHz, DMF-d7): δ 0.91 (t, J = 7.4 Hz, 3H), 1.52 (s, 2H), 2.04 (s, 6H), 2.32 (s, 2H), 2.99 (s, 2H), 3.89 (s, 3H), 4.01 (s, 1H), 7.03 (d, J = 3.6 Hz, 1H), 7.45 (s, 1H), 7.78 (d, J = 7.4 Hz, 1H), 7.90 (s, 1H), 8.17 – 8.21 (m, 1H), 8.64 (d, J = 4.2 Hz, 1H).	14	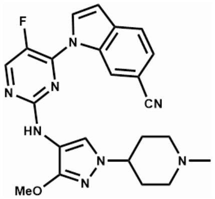	447.3	1H NMR (500 MHz, DMF-d7): δ 1.95 – 2.07 (m, 6H), 2.22 (s, 3H), 2.87 (d, J = 10.7 Hz, 2H), 3.90 (s, 3H), 3.97 (s, 1H), 7.03 (d, J = 3.6 Hz, 1H), 7.63 (dd, J = 8.1, 1.4 Hz, 1H), 7.91 (d, J = 8.1 Hz, 2H), 8.18 (s, 1H), 8.62 (d, J = 4.4 Hz, 1H).
11	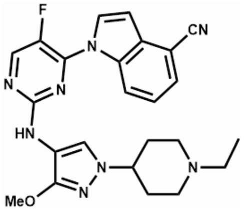	461.2	1H NMR (500 MHz, DMF-d7): δ 1.05 (t, J = 7.2 Hz, 3H), 1.93 – 2.09 (m, 6H), 2.38 (q, J = 7.2 Hz, 2H), 3.01 (d, J = 10.4 Hz, 2H), 3.89 (s, 3H), 3.98 (dt, J = 10.8, 5.8 Hz, 1H), 7.02 (d, J = 3.7 Hz, 1H), 7.45 (d, J = 8.4 Hz, 1H), 7.79 (d, J = 7.4 Hz, 1H), 7.89 (s, 1H), 8.18 (dd, J = 3.6, 2.2 Hz, 1H), 8.63 (d, J = 4.3 Hz, 1H).	15	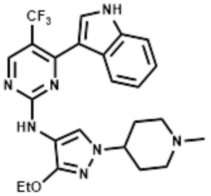	486.2	1H NMR (500 MHz, DMF-d7): δ 1.29 (t, J = 7.0 Hz, 3H), 1.90-2.05 (m, 6H), 2.22 (s, 3H), 2.87 (d, J= 2.8 Hz, 2H), 4.01 (m, 1H), 4.24 (q, J = 7.0 Hz, 2H), 7.02 (s, 1H), 7.12 (s, 1H), 7.94 (s, 1H), 8.26 (s, 1H), 8.47 (s, 1H), 8.57 (s, 1H).
12	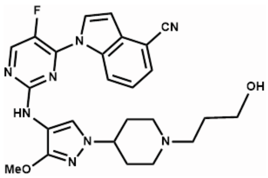	491.3	1H NMR (500 MHz, DMF-d7): δ 1.99 (s, 2H), 2.29 - 2.49 (m, 4H), 3.00 - 3.27 (m, 4H), 3.64 (m, 4H), 3.90 (s, 3H), 4.39 (m, 1H), 7.03 (s, 1H), 7.48 (t, J = 8.0 Hz, 1H), 7.78 (d, J = 7.3 Hz, 1H), 7.95 (s, 1H), 8.19 (s, 1H), 8.64 (d, J = 4.2 Hz, 1H).	16	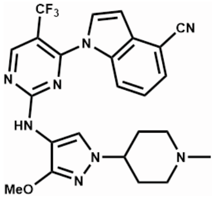	497.1	1H NMR (500 MHz, DMF-d7): δ 2.19 (s, 2H), 2.40 (s, 4H), 2.95 - 3.17 (m, 6H), 3.77 (bs, 1H), 3.83 - 3.94 (m, 6H), 4.23 (m, 1H), 4.48 (m, 1H), 7.41 (t, J = 7.8 Hz, 2H), 7.66 (d, J = 7.3 Hz, 2H), 7.98 (d, J = 8.2 Hz, 2H), 8.04 (m, 2H), 8.77 (s, 2H), 9.20 (bs, 1H), 9.27 (bs, 1H).
13	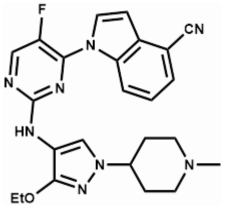	461.2	1H NMR (500 MHz, DMF-d7): δ 1.29 (t, J = 7.0 Hz, 3H), 1.91 – 2.14 (m, 6H), 2.23 (s, 3H), 2.88 (d, J = 11.3 Hz, 2H), 3.95 (m, 1H), 4.24 (q, J = 7.0 Hz, 2H), 7.03 (d, J = 3.6 Hz, 1H), 7.44 (s, 1H), 7.79 (d, J = 7.4 Hz, 1H), 7.90 (s, 1H), 8.18 – 8.2 (m, 1H), 8.64 (d, J = 4.2 Hz, 1H).	17	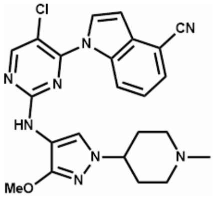	463.3	1H NMR (500 MHz, DMF-d7): δ 2.11 (s, 3H), 2.57 (m, 6H), 3.17 (bs, 2H), 3.89 (s, 3H), 4.08 (m, 1H), 7.42 (t, J = 7.8 Hz, 1H), 7.69 (d, J = 7.3 Hz, 1H), 7.96 (s, 1H), 7.97 (dd, J = 8.3, 1.0 Hz, 1H), 8.33 (s, 1H), 8.49 (s, 1H).

#	Chemical structure	MS (ESI+)	NMR	#	Chemical structure	MS (ESI+)	NMR
18	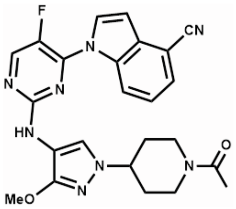	475.3	1H NMR (500 MHz, DMF-d7): δ 1.80 (qd, J = 12.3, 4.5 Hz, 1H), 1.94 (qd, J = 12.2, 11.5, 3.8 Hz, 1H), 2.05 (m, 2H), 2.10 (s, 3H), 2.72 – 2.8 (m, 1H), 3.27 (td, J = 13.8, 13.1, 2.8 Hz, 1H), 3.89 (s, 3H), 4.03 (d, J = 13.9 Hz, 1H), 4.30 (tt, J = 11.3, 4.1 Hz, 1H), 4.58 (d, J = 13.2 Hz, 1H), 7.02 (d, J = 3.6 Hz, 1H), 7.45 (d, J = 7.6 Hz, 1H), 7.78 (d, J = 7.4 Hz, 1H), 7.93 (s, 1H), 8.17 – 8.2 (m, 1H), 8.64 (d, J = 4.2 Hz, 1H).	22	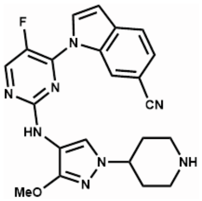	433.1	1H NMR (400 MHz DMSO-d6): δ 9.10 (brs, 1H), 8.73-8.72 (m, 1H), 8.60 (s, 1H), 8.43-8.38 (m, 1H), 8.10 (s, 1H), 7.86-7.80 (m, 2H), 7.60 (d, J = 8.0 Hz, 1H), 6.97 (d, J = 4.0 Hz, 1H), 3.01 (d, J = 8.0 Hz, 1H), 4.32-4.27 (m, 1H), 3.84 (s, 3H), 3.39 (d, J=12.0 Hz, 2H), 3.05-3.02 (m, 2H), 2.17-2.05 (m, 4H).
19	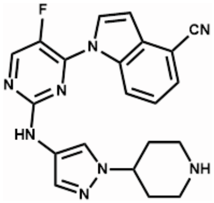	403.1	1H NMR: (400 MHz DMSO-d6): δ 9.84 (s, 1H), 8.71 (s, 2H), 8.40 (s, 1H), 8.12 (s, 1H), 8.11 (s, 1H), 7.79 (d, J = 8.0 Hz, 1H), 7.57 (s, 1H), 7.49-7.45 (m, 1H), 6.99 (s, 1H), 4.44 (s, 1H), 3.41-3.38 (m, 2H), 3.09-3.03 (m, 2H), 2.17-2.04 (m, 4H).	23	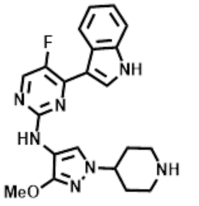	408.1	1H NMR (400 MHz DMSO-d6): δ 11.8 (s, 1H), 8.27 (s, 2H), 8.26 (s, 1H), 7.72 (s, 1H), 7.46 (d, J = 8.0 Hz, 1H), 7.21-7.17 (m, 1H), 7.04 (s, 1H), 4.01-3.99 (m, 1H), 3.77 (s, 3H), 3.03 (d, J = 12 Hz, 2H), 2.56-2.50 (m, 2H), 1.96-1.93 (m, 2H), 1.77-1.73 (m,
20	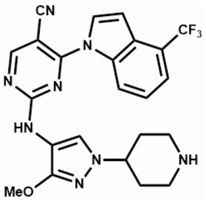	483.0	1H NMR (400 MHz DMSO-d6): δ 8.77 (s, 1H), 8.32-8.69 (dd, 1H), 8.21 (m, 1H), 7.76-7.96 (ds, 1H), 7.60 (m, 1H), 7.35-7.47 (dt, 1H), 6.95 (br s, 1H), 4.26 (m, 1H), 3.94 (m, 3H), 3.50 (m, 3H), 3.15 (m, 2H), 2.19-2.31 (m, 4H).	24	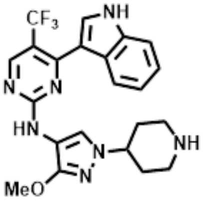	458.1	1H NMR (400 MHz DMSO-d6): δ 11.91 (s, 1H), 9.18-8.98 (m, 1H), 8.61-8.54 (m, 2H), 7.94-7.77 (m, 3H), 7.25 (s, 1H), 7.23 (s, 1H), 7.17-7.10 (m, 2H), 3.78 (s, 3H), 3.40-3.38 (m, 2H), 3.06-3.03 (m, 2H), 2.13-2.09 (m, 4H).
21	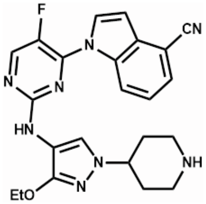	447.1	1H NMR (400 MHz DMSO-d6): δ 8.81-8.79 (m, 2H), 8.61 (s, 1H), 8.46-8.44 (m, 1H), 8.10 (s, 1H), 7.76-7.74 (m, 2H), 7.35 (s, 1H), 6.96(d, J = 3.2 Hz, 1H), 4.31-4.29 (m, 1H), 4.29-4.27 (m, 2H), 3.39 (d, J = 8 Hz, 2H), 3.09-3.00 (m, 2H), 2.15-2.02 (m, 4H), 1.24 (s, 3H).	25	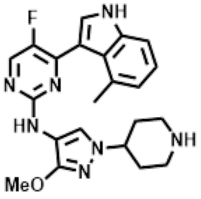	422.1	1H NMR (400 MHz DMSO-d6): δ 11.7 (s, 1H), 8.76-8.74 (m, 1H), 8.41-8.39 (m, 1H), 8.33(s, 2H), 7.74-7.72 (m, 2H), 7.30 (d, J = 8.0 Hz, 1H), 7.09-7.05 (m, 1H), 6.85 (d, J = 8.0 Hz, 1H), 3.77 (s, 3H), 3.38 (d, J = 12.8 Hz, 2H),3.04-3.01 (m, 2H), 2.38 (s,

#	Chemical structure	MS (ESI+)	NMR	#	Chemical structure	MS (ESI+)	NMR
26	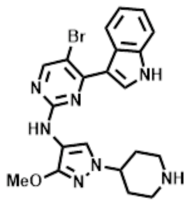	470.0	1H NMR (500 MHz DMSO-d6): δ 8.49 (s, 1H), 8.32 (s, 1H), 8.25 (br s, 2H), 7.68 (s, 1H), 7.43 (m, 1H), 7.25 (t, 1H), 7.05 (m, 1H), 3.95 (m, 1H), 3.77 (s, 3H), 3.38 (d, 2H), 3.02 (d, 2H), 2.55 (m, 2H), 1.93 (d, 2H), 1.72 (m, 2H).	30	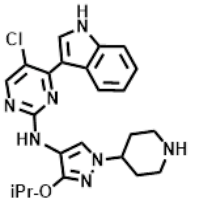	452.1	1H NMR (400 MHz DMSO-d6): δ 11.82 (s, 1H), 8.49 (s, 1H), 8.27-8.31 (m, 2H), 7.69 (s, 1H), 7.45 (d, 1H), 7.18 (t, 1H), 7.01 (br s, 1H), 4.74 (m, 1H), 3.96 (m, 1H), 3.01 (d, 2H), 2.56 (t, 2H), 1.92 (m, 2H), 1.73 (m, 2H), 1.20 (m, 6H).
27	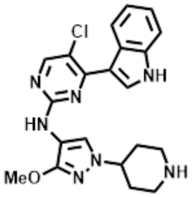	424.1	1H NMR (400 MHz DMSO-d6): δ 11.80 (s, 1H), 8.20-8.70 (m, 4H), 7.70 (s, 1H), 7.37 (d, 1H), 7.12 (t, 1H), 6.95 (br s, 1H), 4.25 (m, 1H), 3.70 (s, 3H), 3.35 (d, 2H), 3.00 (q, 2H), 2.50 (s, 2H), 2.05 (m, 4H).	31	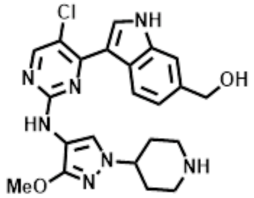	454.0	1H NMR (400 MHz, DMSO-d6) δ ppm 1.62 - 1.85 (m, 2 H) 1.94 (d, J=11.12 Hz, 2 H) 2.53 - 2.62 (m, 2 H) 3.03 (d, J=12.63 Hz, 2 H) 3.78 (s, 3 H) 3.87 - 4.10 (m, 1 H) 4.52 - 4.69 (m, 2 H) 5.15 (br. s., 1 H) 6.99 (br. s., 1 H) 7.41 (s, 1 H) 7.71 (s, 1 H) 8.30 (s, 1 H) 8.35 (br. s., 1 H) 8.43 - 8.52 (m, 1 H) 11.78 (br. s., 1 H)
28	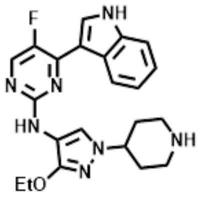	422.1	1H NMR (300 MHz, MeOD-d4): δ ppm 8.38 (dd, J=8.01, 2.92 Hz, 1 H), 8.10 (d, J=3.01 Hz, 1 H), 8.04 (d, J=4.52 Hz, 1 H), 7.69 (s, 1 H), 7.38 (d, J=8.10 Hz, 1 H), 7.11 - 7.24 (m, 1 H), 6.93 - 7.09 (m, 1 H), 4.21 - 4.34 (m, 1H), 4.05 - 4.20 (m, 2 H), 3.43 - 3.61 (m, 2 H), 3.05 - 3.16 (m, 2 H), 2.08 - 2.37 (m, 4 H), 1.22 (t, J=6.97 Hz, 3H)	32	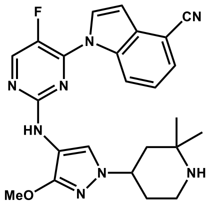	461.3	1H NMR: (400 MHz DMSO-d6): δ 8.82 (br s, 1H), 8.77 (s, 1H), 8.09 (s, 1H), 7.74 (m, 2H), 7.34 (br s, 1H), 6.96 (s, 1H), 4.17 (m, 1H), 3.79 (s, 3H), 2.80 (m, 2H), 1.75-1.87 (dd, 2H), 1.51-1.61 (m, 3H), 1.10 (s, 3H), 1.06 (s, 3H).
29	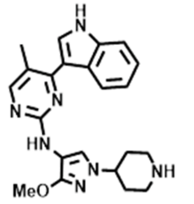	404.0	1H NMR (300 MHz, MeOD-d4): δ 8.31 (d, 1H), 8.13 (s, 1H), 7.82 (d, 2H), 7.47 (d, 1H), 7.09-7.48 (m, 3H), 3.90-4.05 (m, 4H), 3.35 (m, 2H), 3.13 (d, 2H), 2.69 (t, 2H), 2.38 (s, 3H), 1.76-2.07 (m, 4H).	33	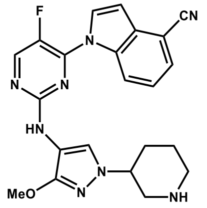	433.1	1H NMR: (400 MHz DMSO-d6): δ 8.81 (br s, 1H), 8.60 (s, 1H), 8.09 (s, 1H), 7.75 (m, 2H), 7.33 (br s, 1H), 6.96 (s, 1H), 3.90 (m, 1H), 3.79 (s, 3H), 3.12 (d, 1H), 2.84 (d, 1H), 2.65 (t, 1H), 2.44 (t, 1H), 2.01 (m, 1H), 1.70-1.81 (m, 2H), 1.45 (m, 1H).
34	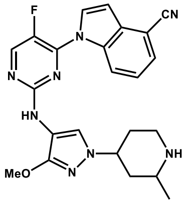	447.2	1H NMR: (400 MHz DMSO-d6): δ 8.56 (m, 3H), 8.07 (m, 1H), 7.70 (m, 2H), 7.35 (t, 1H), 6.94 (m, 1H), 3.99 (m, 1H), 3.82 (s, 3H), 3.05 (m, 2H), 2.66 (m, 2H), 1.93 (t, 2H), 1.68 (m, 1H), 1.36 (m, 1H), 1.05 (d, 3H).	35	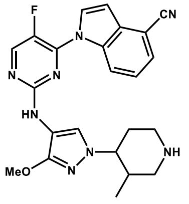	447.1	1H NMR: (400 MHz DMSO-d6): δ 8.55 (br s, 3H), 8.05 (s, 1H), 7.64-7.71 (m, 2H), 7.33 (m, 1H), 6.93 (br s, 1H), 4.16 (br s, 1H), 3.83 (br s, 3H), 3.15 (br s, 1H), 2.80 (m, 2H), 2.65 (m, 1H), 2.21 (br s, 1H), 1.97 (m, 1H), 1.74 (m, 1H), 0.71 (d, 3H).

Table S2
Gal9 response from functionally selected compounds

EEE	Gal9 response compared to reference (Calculated mean from >3 independent exp.)								
	(µM)	16HBE	HEK293	HeLa	HepG2	HuH7	NCI-H358	SHSY5Y	U2OS
4	0.31	0.021	0.027	0.005	0.010	0.012	0.020	0.016	0.014
	0.62	0.039	0.243	0.011	0.014	0.033	0.026	0.315	0.019
	1.25	0.395	0.648	0.246	0.030	0.199	0.280	0.678	0.083
	2.5	0.626	0.678	0.481	0.410	0.245	0.926	0.499	0.332
	5	0.501	0.396	0.168	0.444	0.189	0.647	0.389	0.572
	10	0.146	0.222	0.058	0.480	0.045	0.293	0.026	0.561
8	0.31	0.011	0.027	0.004	0.007	0.010	0.017	0.014	0.012
	0.62	0.035	0.052	0.009	0.008	0.016	0.032	0.082	0.013
	1.25	0.180	0.309	0.105	0.011	0.078	0.148	0.694	0.029
	2.5	0.503	0.657	0.319	0.075	0.313	0.553	0.629	0.234
	5	0.638	0.605	0.459	0.511	0.228	0.623	0.567	0.490
	10	0.492	0.329	0.198	0.529	0.160	0.457	0.278	0.563
9	0.31	0.010	0.028	0.005	0.009	0.012	0.018	0.013	0.012
	0.62	0.013	0.032	0.005	0.009	0.009	0.020	0.016	0.013
	1.25	0.042	0.071	0.007	0.007	0.019	0.031	0.117	0.018
	2.5	0.209	0.396	0.110	0.011	0.083	0.212	0.756	0.055
	5	0.533	0.712	0.393	0.060	0.220	0.569	0.780	0.254
	10	0.691	0.639	0.495	0.564	0.366	0.627	0.696	0.502
10	0.31	0.010	0.023	0.005	0.008	0.011	0.017	0.015	0.011
	0.62	0.011	0.026	0.005	0.007	0.009	0.016	0.024	0.012
	1.25	0.028	0.051	0.005	0.007	0.016	0.031	0.123	0.016
	2.5	0.183	0.379	0.145	0.008	0.121	0.194	0.877	0.076
	5	0.522	0.672	0.463	0.026	0.252	0.633	0.887	0.260
	10	0.746	0.647	0.588	0.371	0.414	0.662	0.717	0.512
11	0.31	0.013	0.027	0.005	0.009	0.009	0.016	0.014	0.012
	0.62	0.013	0.022	0.004	0.008	0.008	0.020	0.018	0.015
	1.25	0.056	0.106	0.013	0.007	0.023	0.036	0.385	0.018
	2.5	0.167	0.438	0.253	0.019	0.154	0.143	0.801	0.048
	5	0.507	0.779	0.553	0.163	0.258	0.614	0.691	0.317
	10	0.623	0.493	0.370	0.568	0.222	0.576	0.469	0.483
12	0.31	0.014	0.023	0.005	0.008	0.014	0.018	0.014	0.013
	0.62	0.063	0.122	0.015	0.010	0.031	0.032	0.271	0.017
	1.25	0.180	0.432	0.195	0.038	0.143	0.128	0.742	0.057
	2.5	0.658	0.716	0.550	0.146	0.357	0.574	0.706	0.299
	5	0.681	0.604	0.394	0.517	0.232	0.607	0.575	0.511
	10	0.421	0.273	0.118	0.574	0.103	0.422	0.177	0.588
13	0.31	0.009	0.018	0.005	0.008	0.010	0.019	0.014	0.014
	0.62	0.011	0.024	0.004	0.007	0.007	0.018	0.015	0.011
	1.25	0.010	0.021	0.004	0.008	0.011	0.013	0.012	0.010
	2.5	0.013	0.020	0.003	0.009	0.015	0.014	0.013	0.014
	5	0.044	0.032	0.007	0.006	0.012	0.015	0.044	0.023
	10	0.306	0.097	0.023	0.007	0.046	0.038	0.421	0.072

EEE	Gal9 response compared to reference (Calculated mean from >3 independent exp.)								
	(µM)	16HBE	HEK293	HeLa	HepG2	HuH7	NCI-H358	SHSY5Y	U2OS
14	0.31	0.010	0.023	0.005	0.008	0.009	0.015	0.013	0.013
	0.62	0.013	0.019	0.005	0.005	0.007	0.019	0.014	0.013
	1.25	0.014	0.018	0.004	0.005	0.010	0.019	0.017	0.015
	2.5	0.031	0.035	0.006	0.005	0.016	0.026	0.083	0.023
	5	0.118	0.123	0.035	0.015	0.047	0.073	0.403	0.058
	10	0.421	0.443	0.213	0.155	0.280	0.457	0.645	0.232
15	0.31	0.006	0.020	0.004	0.008	0.008	0.013	0.014	0.010
	0.62	0.007	0.019	0.004	0.008	0.007	0.016	0.017	0.009
	1.25	0.007	0.015	0.004	0.009	0.008	0.017	0.014	0.010
	2.5	0.008	0.015	0.005	0.007	0.009	0.015	0.016	0.009
	5	0.010	0.018	0.004	0.007	0.009	0.016	0.012	0.014
	10	0.034	0.022	0.003	0.010	0.011	0.011	0.012	0.014
16	0.31	0.008	0.023	0.004	0.010	0.009	0.014	0.014	0.010
	0.62	0.009	0.020	0.004	0.007	0.007	0.017	0.014	0.011
	1.25	0.008	0.019	0.003	0.008	0.008	0.014	0.012	0.010
	2.5	0.007	0.022	0.004	0.008	0.012	0.018	0.012	0.010
	5	0.011	0.024	0.004	0.009	0.008	0.014	0.012	0.010
	10	0.129	0.121	0.005	0.010	0.022	0.019	0.042	0.024
17	0.31	0.008	0.023	0.005	0.009	0.007	0.016	0.014	0.012
	0.62	0.010	0.022	0.004	0.008	0.007	0.019	0.013	0.011
	1.25	0.008	0.019	0.004	0.008	0.010	0.014	0.012	0.013
	2.5	0.010	0.019	0.004	0.011	0.017	0.018	0.014	0.015
	5	0.048	0.096	0.012	0.010	0.021	0.028	0.176	0.021
	10	0.488	0.437	0.201	0.030	0.132	0.303	0.610	0.155
18	0.31	0.007	0.022	0.004	0.007	0.008	0.012	0.012	0.007
	0.62	0.008	0.025	0.003	0.006	0.009	0.014	0.013	0.008
	1.25	0.007	0.019	0.003	0.006	0.010	0.014	0.011	0.009
	2.5	0.008	0.019	0.003	0.007	0.011	0.014	0.012	0.008
	5	0.009	0.027	0.003	0.005	0.007	0.016	0.013	0.006
	10	0.010	0.016	0.006	0.006	0.012	0.017	0.011	0.008
19	0.31	0.009	0.026	0.003	0.005	0.009	0.016	0.011	0.009
	0.62	0.011	0.016	0.003	0.006	0.009	0.021	0.015	0.009
	1.25	0.012	0.020	0.003	0.006	0.013	0.010	0.012	0.013
	2.5	0.020	0.024	0.005	0.006	0.014	0.015	0.051	0.015
	5	0.115	0.151	0.045	0.022	0.061	0.070	0.243	0.070
	10	0.491	0.396	0.337	0.308	0.284	0.451	0.511	0.403
20	0.31	0.009	0.024	0.004	0.006	0.010	0.015	0.011	0.010
	0.62	0.012	0.018	0.005	0.007	0.008	0.017	0.011	0.013
	1.25	0.014	0.027	0.005	0.007	0.014	0.014	0.012	0.015
	2.5	0.064	0.390	0.014	0.011	0.032	0.040	0.262	0.057
	5	0.354	0.575	0.323	0.048	0.126	0.412	0.606	0.254
	10	0.413	0.540	0.561	0.479	0.506	0.461	0.319	0.514

EEE	Gal9 response compared to reference (Calculated mean from >3 independent exp.)								
	(μM)	16HBE	HEK293	HeLa	HepG2	HuH7	NCI-H358	SHSY5Y	U2OS
21	0.31	0.011	0.022	0.004	0.008	0.011	0.017	0.012	0.012
	0.62	0.010	0.021	0.003	0.006	0.009	0.014	0.014	0.012
	1.25	0.014	0.018	0.004	0.007	0.010	0.011	0.015	0.019
	2.5	0.119	0.095	0.009	0.007	0.020	0.015	0.127	0.026
	5	0.284	0.406	0.083	0.013	0.086	0.096	0.595	0.167
	10	0.602	0.498	0.462	0.424	0.411	0.520	0.645	0.437
22	0.31	0.009	0.020	0.004	0.009	0.010	0.015	0.014	0.011
	0.62	0.011	0.019	0.004	0.006	0.010	0.017	0.014	0.010
	1.25	0.018	0.021	0.003	0.006	0.012	0.011	0.020	0.014
	2.5	0.155	0.219	0.029	0.006	0.055	0.042	0.528	0.058
	5	0.477	0.573	0.249	0.269	0.285	0.570	0.690	0.235
	10	0.722	0.517	0.527	0.544	0.410	0.681	0.673	0.493
23	0.31	0.008	0.019	0.004	0.006	0.007	0.013	0.013	0.011
	0.62	0.010	0.015	0.005	0.006	0.008	0.015	0.015	0.012
	1.25	0.010	0.015	0.004	0.007	0.009	0.012	0.013	0.015
	2.5	0.012	0.016	0.005	0.005	0.009	0.017	0.013	0.015
	5	0.017	0.025	0.005	0.004	0.010	0.017	0.017	0.012
	10	0.032	0.042	0.008	0.005	0.011	0.021	0.087	0.017
24	0.31	0.010	0.024	0.005	0.010	0.010	0.019	0.016	0.014
	0.62	0.011	0.019	0.005	0.009	0.010	0.018	0.015	0.015
	1.25	0.011	0.014	0.004	0.009	0.011	0.016	0.010	0.014
	2.5	0.014	0.017	0.004	0.008	0.016	0.015	0.013	0.015
	5	0.035	0.100	0.008	0.008	0.019	0.021	0.070	0.026
	10	0.335	0.464	0.207	0.104	0.174	0.115	0.703	0.166
25	0.31	0.009	0.031	0.005	0.009	0.010	0.016	0.014	0.010
	0.62	0.011	0.024	0.004	0.007	0.006	0.016	0.013	0.010
	1.25	0.010	0.020	0.004	0.009	0.012	0.018	0.012	0.009
	2.5	0.013	0.020	0.005	0.010	0.013	0.020	0.011	0.010
	5	0.015	0.025	0.005	0.008	0.013	0.016	0.010	0.014
	10	0.071	0.392	0.009	0.012	0.031	0.021	0.348	0.017

Table S3
Functional 705-SSO delivery in HeLa_Luc705

Luciferase expression compared to reference
(Mean from >3 independent experiments using HeLa-Luc705 + **2 µM** SSO)

EEE		4	8	9	10	11	12	13
(µM)	1-1.25	80.967	0.687	ND	ND	ND	ND	0.743
	2.5 - 3	86.370	102.187	0.692	0.565	2.093	1.720	0.692
	5	0.020	52.876	ND	ND	ND	ND	1.314

EEE		14	15	16	17	18	19	20
(µM)	1-1.25	ND	ND	ND	ND	0.690	0.787	0.657
	2.5 - 3	0.590	0.652	0.810	0.752	0.930	0.817	4.400
	5	ND	ND	ND	ND	7.102	0.888	2.255

EEE		21	22	23	24	25
(µM)	1-1.25	0.668	0.707	0.775	0.741	0.678
	2.5 - 3	7.590	2.447	1.207	1.920	7.890
	5	0.674	0.706	65.259	7.300	1.699

(Mean from >3 independent experiments using HeLa-Luc705 + **1 µM** SSO)

EEE		4	8	32	33	34	35
(µM)	0.5	3.610	0.717	3.743	0.979	1.686	1.095
	1-1.25	11.217	0.677	18.028	0.995	6.877	1.101
	2	139.083	2.253	115.294	1.980	29.352	1.804
	2.5 - 3	106.930	4.247	133.534	4.714	51.794	2.812
	4	39.821	11.227	111.401	8.046	67.021	4.870
	5	13.700	31.147	66.337	27.418	ND	9.588

SYNTHETIC PROCEDURES

General procedure as illustrated for preparation of Compound 4

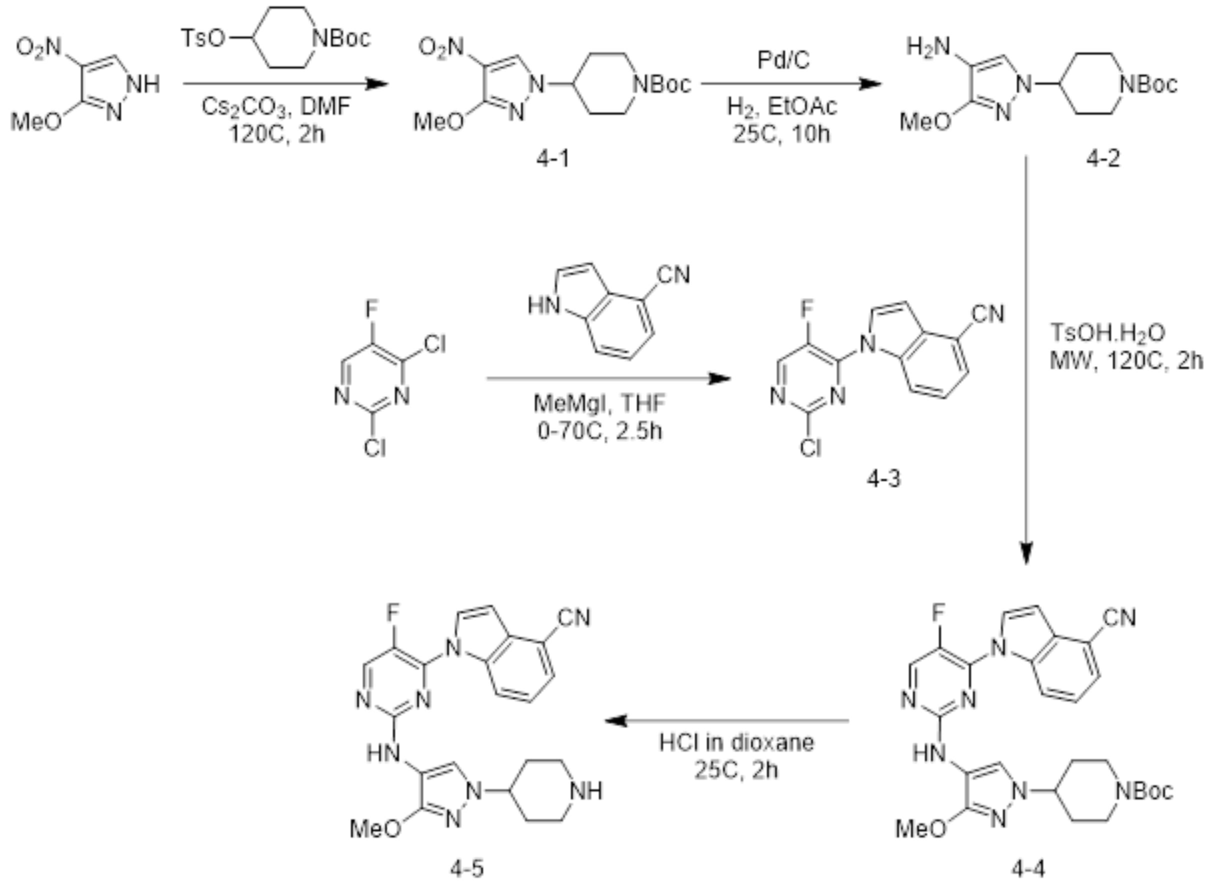

tert-butyl 4-(3-methoxy-4-nitro-1*H*-pyrazol-1-yl)piperidine-1-carboxylate 4-1

3-methoxy-4-nitro-1H-pyrazole (9.00 g, 62.8 mmol) and tert-butyl 4-(tosyloxy)piperidine-1-carboxylate (28.8 g, 81.1 mmol, 1.29 *eq*) were solubilized in DMF (63 mL). Cs_2CO_3 (30.5 g, 93.7 mmol, 1.49 *eq*) was added and the reaction mixture was degassed 3 times with nitrogen and subsequently stirred at 120 °C for 2 hours. The mixture was then neutralized with HCl (1 M) and extracted with DCM (200 mL x 3). The organic layer was washed with brine (100 mL x 2), dried over magnesium sulfate then concentrated in vacuo. The residue was purified by column chromatography (SiO_2, Petroleum ether/Ethyl acetate=30/1 to 3/1) to yield compound 4-1 (18.1 g, 70.5% yield, 80.0% purity) as a yellow solid.

^{1}H NMR: 400 MHz DMSO-*d*$_6$
δ 8.746 (s, 1H), 7.94 (s, 1H), 4.69-4.68 (m, 1H), 4.31-4.26 (m, 2H), 4.05 (s, 3H), 3.33 (s, 4H), 2.88 (s, 3H), 2.72 (s, 1H), 2.01-1.98 (m, 2H), 1.81-1.74 (m, 2H), 1.40 (s, 9H).

***tert*-butyl 4-(4-amino-3-methoxy-1*H*-pyrazol-1-yl)piperidine-1-carboxylate 4-2**

Compound 4-1 (17.0 g, 52.0 mmol, 1.00 *eq*) was solubilized in EtOAc (110 mL) in a round-bottom flask then charged with Pd/C 10% (1.70 g, 52.0 mmol, 1.00 *eq*). The reaction flask was then purged with nitrogen and filled with H_2 (15 Psi), and the mixture was stirred at 25 °C for 10 hours. The suspension was subsequently filtered through celite, washed with EtOAc (150 mL x 3) and the filtrate was concentrated in vacuo. The residue was purified by column chromatography (SiO_2, Petroleum ether/Ethyl acetate=30/1 to 3/1) to yield compound 4-2 (10.9 g, 70.6% yield) as a black-blue oil.

^{1}H NMR: 400 MHz DMSO-d_6
δ 7.00 (s, 1H), 4.01-3.94 (m, 3H), 3.52 (s, 3H), 2.88-2.73 (m, 2H), 1.88-1.84 (m, 2H), 1.66-1.60 (m, 2H), 1.40 (s, 9H)

1-(2-chloro-5-fluoropyrimidin-4-yl)-1*H*-indole-4-carbonitrile 4-3

1H-indole-4-carbonitrile (6.81 g, 47.9 mmol) was solubilized in THF (300 mL) in a round-bottom flask under nitrogen, at 0 °C, before methylmagnesium iodide (3 M in THF, 15.97 mL, 1.00 *eq*) was added dropwise. The resulting solution was stirred at 0 °C for 30 min, before 2,4-dichloro-5-fluoropyrimidine (8.00 g, 47.9 mmol, 1.00 *eq*) was added. The reaction mixture was then allowed to warm up to room temperature and stirred at 70 °C for 15 hours. Saturated aqueous NH_4Cl solution was subsequently added until pH 7-8 and the mixture was extracted with EtOAc (300 mL x 3). The organic layer was washed with brine (200 mL x 2), dried over magnesium sulfate then concentrated in vacuo. The residue was purified by column chromatography (SiO_2, Petroleum ether/Ethyl acetate=30/1 to 3/1) to yield compound 4-3 (5.00 g, 19.1% yield, 50% purity) as a yellow solid.

***tert*-butyl 4-(4-((4-(4-cyano-1*H*-indol-1-yl)-5-fluoropyrimidin-2-yl)amino)-3-methoxy-1*H*-pyrazol-1-yl)piperidine-1-carboxylate 4-4**

Compound 4-3 (399 mg, 1.47 mmol) and compound 4-2 (436 mg, 1.47 mmol, 1 *eq*) were charged in i-PrOH (7.00 mL) in a microwave vial. The tube was heated in a microwave oven at 120 °C for 2 hours. Saturated aqueous $NaHCO_3$ solution was then added until neutral pH. The resulting mixture was extracted with EtOAc (100 mL x 3) and the organic layer was washed with brine (100 mL x 2), dried over magnesium sulfate and concentrated in vacuo to yield compound 4-4 (0.6 g, crude) as a white solid, used as such in the next reaction.

1-(5-fluoro-2-((3-methoxy-1-(piperidin-4-yl)-1*H*-pyrazol-4-yl)amino)pyrimidin-4-yl)-1*H*-indole-4-carbonitrile 4-5

Compound 4-4 (0.60 g, 1.13 mmol) was dissolved in HCl/dioxane (4 M, 4.20 mL, 14.9 *eq*) and stirred at 25°C for 2 hours. Saturated aqueous $NaHCO_3$ solution (50.0 mL) was then carefully

added. The mixture was extracted with EtOAc (100.0 mL x 2) and the organic layer was dried over magnesium sulfate and concentrated in vacuo. The residue was purified by preparative HPLC (column: Phenomenex Gemini-NX 80x40mmx3um; mobile phase: [water (10mM NH_4HCO_3)-ACN];B%: 20%-50%,8min), yielding compound 4-5 (0.05 g, 10.0% yield, 98.3% purity) as a yellow solid.

1H NMR: 400 MHz DMSO-*d*$_6$
Compound 4: δ 8.81 (brs, 1H), 8.60 (d, *J* = 4.0 Hz, 1H), 8.08 (s, 1H), 7.73 (s, 1H), 7.32 (brs, 1H), 6.95 (d, *J* = 4.0 Hz, 1H), 3.99-3.94 (m, 1H), 3.79 (s, 1H), 3.01 (d, *J* = 8.0 Hz, 1H), 2.57-2.54 (m, 2H), 1.90-1.87 (m, 2H), 1.71-1.68 (m, 2H).
